# Instruments measuring evidence-based practice behavior, attitudes, and self-efficacy among healthcare professionals: a systematic review of measurement properties

**DOI:** 10.1186/s13012-023-01301-3

**Published:** 2023-09-13

**Authors:** Nils Gunnar Landsverk, Nina Rydland Olsen, Therese Brovold

**Affiliations:** 1 Department of Rehabilitation Science and Health Technology, Faculty of Health Science, Oslo Metropolitan University, Oslo, Norway; 2 Department of Health and Functioning, Faculty of Health and Social Sciences, Western Norway University of Applied Sciences, Bergen, Norway

**Keywords:** Evidence-based practice, Healthcare professional, Attitudes, Self-efficacy, Behavior, Development, Instrument, Validity, Reliability, Measurement error

## Abstract

**Background:**

Evidence-based practice (EBP) is well known to most healthcare professionals. Implementing EBP in clinical practice is a complex process that can be challenging and slow. Lack of EBP knowledge, skills, attitudes, self-efficacy, and behavior can be essential barriers that should be measured using valid and reliable instruments for the population in question. Results from previous systematic reviews show that information regarding high-quality instruments that measure EBP attitudes, behavior, and self-efficacy in various healthcare disciplines need to be improved. This systematic review aimed to summarize the measurement properties of existing instruments that measure healthcare professionals’ EBP attitudes, behaviors, and self-efficacy.

**Methods:**

We included studies that reported measurement properties of instruments that measure healthcare professionals’ EBP attitudes, behaviors, and self-efficacy. Medline, Embase, PsycINFO, HaPI, AMED via Ovid, and Cinahl via Ebscohost were searched in October 2020. The search was updated in December 2022. The measurement properties extracted included data on the item development process, content validity, structural validity, internal consistency, reliability, and measurement error. The quality assessment, rating of measurement properties, synthesis, and modified grading of the evidence were conducted in accordance with the COSMIN methodology for systematic reviews.

**Results:**

Thirty-four instruments that measure healthcare professionals’ EBP attitudes, behaviors or self-efficacy were identified. Seventeen of the 34 were validated in two or more healthcare disciplines. Nurses were most frequently represented (*n* = 53). Despite the varying quality of instrument development and content validity studies, most instruments received sufficient ( +) ratings on content validity, with the quality of evidence graded as “very low” in most cases. Structural validity and internal consistency were the measurement properties most often assessed, and reliability and measurement error were most rarely assessed. The quality assessment results and overall rating of these measurement properties varied, but the quality of evidence was generally graded higher for these properties than for content validity.

**Conclusions:**

Based on the summarized results, the constructs, and the population of interest, several instruments can be recommended for use in various healthcare disciplines. However, future studies should strive to use qualitative methods to further develop existing EBP instruments and involve the target population.

**Trial registration:**

This review is registered in PROSPERO. CRD42020196009. Available from: https://www.crd.york.ac.uk/prospero/display_record.php?ID=CRD42020196009

**Supplementary Information:**

The online version contains supplementary material available at 10.1186/s13012-023-01301-3.

Contributions to the literature • Evidence-based practice (EBP) is well-known to most healthcare professionals and has become the gold standard in healthcare. To implement EBP sufficiently among healthcare personnel, we need valid and reliable instruments to measure EBP attitudes, self-efficacy, and behavior.

• This study identified several instruments that can be recommended in different healthcare disciplines, adding knowledge that could help choose an instrument for use in future studies implementing EBP in clinical practice.

• Our findings also underpin the importance of involving the target population and using qualitative methods when developing new EBP instruments or adapting existing EBP instruments measuring EBP attitudes, self-efficacy, and behavior.

## Background

Evidence-based practice (EBP) is well known to most healthcare professionals. EBP refers to the integration of the best available research evidence with clinical expertise and patient characteristics and preferences [1]. EBP has become the gold standard in healthcare. Implementing EBP in clinical practice is associated with high-quality care, such as improved patient outcomes, reduced costs, and increased job satisfaction [2–6].

Implementing EBP in clinical practice is a complex process that is challenging and slow [3, 7]. The implementation of EBP can be hindered by barriers, including organizational, cultural, or clinician-related factors. At clinician-related level, research shows that a lack of EBP knowledge, insufficient skills, negative attitudes, low self-efficacy, and lack of EBP behaviors can be essential barriers [8, 9]. The different steps of the EBP process require that healthcare professionals understand the concepts of EBP (knowledge) and have the practical skills to do EBP activities, such as searching electronic databases or using critical appraisal tools (skills) [1, 10]. Further, the healthcare professionals’ confidence in their ability to perform EBP activities (self-efficacy), and their beliefs in the positive benefits of EBP (attitudes), are known to be associated with the likelihood of EBP being successfully implemented in clinical practice (behavior) [10–12].

Strategies to improve EBP implementation should be tailored based on the healthcare professionals' perceived barriers [13–15]. However, many healthcare institutions are unaware of potential barriers that could be related to EBP knowledge, skills, attitudes, self-efficacy, and behavior among their workers [7]. These EBP constructs should be measured using valid and reliable instruments for the population in question [10]. Former systematic reviews have recommended using and further developing instruments such as the Fresno test as a measure of EBP knowledge and skills across healthcare disciplines based on existing documentation of validity and reliability on this instrument [7, 10, 16–19]. However, such clear recommendations do not exist for instruments that measure EBP attitudes, self-efficacy, and behavior.

Although several reviews have assessed instruments that measure EBP attitudes, behavior or self-efficacy [20–25], none focused on all three constructs, nor did they include studies across different healthcare disciplines. For instance, Hoegen et al. [20] included only self-efficacy instruments, and Oude Rengerink et al. [21] included only instruments measuring EBP behavior. The reviews from Belita et al. [25], Hoegen et al. [20], Leung et al. [22], Fernández-Domínguez et al. [24], and Buchanan et al. [23] included studies from one specific healthcare discipline only. A review focusing on all three constructs are needed, given the known associations between these constructs [10–12]. In addition, including studies across different healthcare disciplines could make the review more relevant for researchers targeting an interdisciplinary population.

Methodological limitations across several previous reviews may influence whether one can trust existing recommendations. Although most of the reviews evaluated the included instruments’ measurement properties [20, 22–25], only Hoegen et al. [20] and Buchanan et al. [23] assessed the risk of bias in the studies included. In addition, none of the reviews rated the quality of the instruments’ development processes in detail [26], and only Hoegen et al. [20] graded the quality of the total body of evidence per instrument using a modified GRADE (Grading of Recommendations Assessment, Development, and Evaluation) approach.

In short, the results from previous systematic reviews show that information regarding high-quality instruments that measure EBP attitudes, behavior, and self-efficacy among various healthcare disciplines is still lacking. A methodologically sound review is needed to evaluate whether instruments that measure EBP attitudes, behavior, and self-efficacy can be recommended across different healthcare disciplines.

### Objectives

This systematic review aimed to summarize the measurement properties of existing instruments that measure healthcare professionals’ EBP attitudes, behaviors, and self-efficacy. We aimed to review the included studies’ methodological quality systematically and to evaluate the instruments’ development process, content validity, structural validity, internal consistency, reliability, and measurement error in accordance with the Consensus‐based standards for the selection of health measurement instruments (COSMIN) methodology for systematic reviews [26–28].

## Methods

This systematic review was conducted and reported following the PRISMA 2020 checklist (Preferred Reporting Items for Systematic Reviews and Meta-Analyses) [29]. The checklist is presented in Additional file 5.

### Eligibility criteria

Studies were included if they met the following criteria: included healthcare professionals (e.g., nurses, physiotherapists, occupational therapists, medical doctors, psychologists, dentists, pharmacists, social workers) from primary or specialized healthcare; reported findings from the development of or the validation process of self-reported EBP instruments; described instruments measuring EBP attitudes, behavior or self-efficacy, or a combination of these EBP constructs; used a quantitative or qualitative design; and published in English or a Scandinavian language.

Studies were excluded based on the following criteria: included undergraduate students or samples from school setting; did not present any psychometric properties; focused on evidence-based diagnosis or management rather than on EBP in general; focused on the effect of implementation strategies rather than on the development or validation of an instrument; and described instruments measuring only EBP knowledge or skills.

### Information sources

The following databases were included in two searches conducted in October 2020 and December 2022: MEDLINE, Embase, PsycINFO, HaPI, and AMED via Ovid, Cinahl via Ebscohost, Web of Science, and Google Scholar. In addition, we used other sources to supplement the search in the electronic databases, including searches in the reference lists of included studies and searches for gray literature. The gray literature search included targeted website searches, advanced Google searches, gray literature databases and catalogs of gray literature, and searches for theses, dissertations, and conference proceedings. The search strategy is described in Additional file 1.

### Search strategy

The search strategy was developed in consultation with and conducted by two academic librarians from OsloMet University Library. The search included terms that were related to or described the nature of the objectives and the inclusion criteria and were built around the following five elements: (1) evidence-based practice, (2) health personnel, (3) measurement and instruments, (4) psychometrics, and (5) behavior, attitude, self-efficacy.

### Selection process

Titles and abstracts of studies retrieved in the search were screened independently by two review team members (NGL and TB). The studies that potentially met the inclusion criteria were identified, and the full texts of these studies were assessed for eligibility by two review members (NGL and TB). In cases of uncertainty regarding inclusion of studies, a third review member was consulted to reach a consensus (NRO). The screening and full-text assessment were conducted using Covidence systematic review software [30].

### Data extraction

Data extraction was piloted on four references using a standard form completed by the first author and checked by two other review members (NRO and TB). The following data on study characteristics were extracted: author(s), publication year, title, aim, study country, study design, sample size, response rate, population/healthcare discipline description, and study setting. Data on the instruments were also extracted, including instrument name, EBP constructs measured (EBP attitudes, behaviors, and self-efficacy), theoretical framework used, EBP steps covered (ask, search, appraise, integrate, evaluate), number of items, number of subscales, scale type, instrument language, availability of questions, and translation procedure. Data on the EBP constructs measured were based on definitions from the CREATE framework (Classification Rubric for Evidence-Based Practice Assessment Tools in Education) [10]. In line with the CREATE framework, we defined the EBP constructs as follows: (1) EBP attitudes: the values ascribed to the importance and usefulness of EBP in clinical decision-making, (2) EBP self-efficacy*:* the judgment regarding one’s ability to perform a specific EBP activity, and (3) EBP behavior*:* what is being done in practice. Finally, data on the instrument’s measurement properties were extracted, including data on the item development process, content validity, structural validity, internal consistency, reliability, and measurement error. Data extraction on all items was performed by the first author.

### Study quality assessment

The review members (NGL, TB, and NRO) independently assessed the methodological quality of each study, using the COSMIN risk of bias checklist for systematic reviews of self-reported outcome measures [27]. Two members reviewed each study. The COSMIN checklist contains standards referring to the quality of each measurement property of interest in this review [27, 31]. The review members followed COSMIN’s four-point rating system, rating the standard of each property as “very good,” “adequate,” “doubtful,” or “inadequate” [27]. The lowest rating per measurement property was used to determine the risk of bias on that particular property, following the “worst score counts” principle [32]. After all the studies were assessed separately by the review members, a consensus on the risk of bias ratings was reached in face-to-face meetings.

### Synthesis methods

The evidence synthesis process was conducted using the COSMIN methodology [26, 31]. The review members rated all the results separately, and a consensus was reached in face-to-face meetings. Instrument development and content validity studies were rated independently by the review authors according to criteria determining whether the instrument’s items adequately reflected the construct to be measured [26]. These included five criteria on relevance, one criterion on comprehensiveness, and four criteria on comprehensibility [26]. The relevance, comprehensiveness, and comprehensibility per study were rated as *sufficient* (+), *insufficient* (−), inconsistent (+ / −) or *indeterminate* (?). The reviewers also rated the instruments themselves. An overall rating was given for the relevance, comprehensibility, and comprehensiveness of each instrument, combining the results from the ratings of each study with the reviewers’ ratings on the same instrument. The overall rating could not be *indeterminate* (?) because the reviewers’ ratings were always available [26]. The assessment of instrument development studies included evaluating the methods used to generate items (concept elicitation) and the methods used to test the new instrument [26]. COSMIN recommends using qualitative methods, involving the target population, when developing instrument items [26].

Results for structural validity, internal consistency, reliability, and measurement error were rated independently against the COSMIN criteria for good measurement properties [28, 33, 34]. Each measurement property was rated as *sufficient* ( +), *insufficient* ( −) or *indeterminate (?).* To conclude each instrument, an overall rating was given for each instrument per property by jointly assessing the results from all the available studies. If the results per property per instrument were consistent, the results could be qualitatively summarized and rated overall as *sufficient* ( +), *insufficient* ( −), *inconsistent* (+ / −) or *indeterminate* (?). More information on the COSMIN criteria for good measurement properties is provided in Additional file 2. Details on the COSMIN guideline for assessing and calculating structural validity, internal consistency, reliability, and measurement error can be found elsewhere (28, 31).

### Certainty assessment

After rating the summarized results per instrument per property against the criteria for good measurement properties, we graded the quality of this evidence to indicate whether or not the overall ratings were trustworthy. The GRADE approach is used to grade the quality of evidence on four levels: *high*, *moderate*, *low,* and *very low* [35]. We used the COSMIN’s modified GRADE approach, where four of the five original GRADE factors are adopted for grading the quality of evidence in systematic reviews of patient-reported outcome measures [28]. We downgraded the quality of evidence when there was concern about the results related to any of these four factors: *risk of bias*, *inconsistency*, *imprecision* or *indirectness*. Further details on the modified GRADE approach are provided in “COSMIN methodology for systematic reviews of Patient-Reported Outcome Measures (PROMs)—user manual” [28]. The quality of evidence was not graded in cases where the overall rating for a measurement property was indeterminate (?) [28]. Nor was evidence graded in cases where the overall ratings were inconsistent and impossible to summarize [31].

## Results

### Study selection

**Fig. 1:**
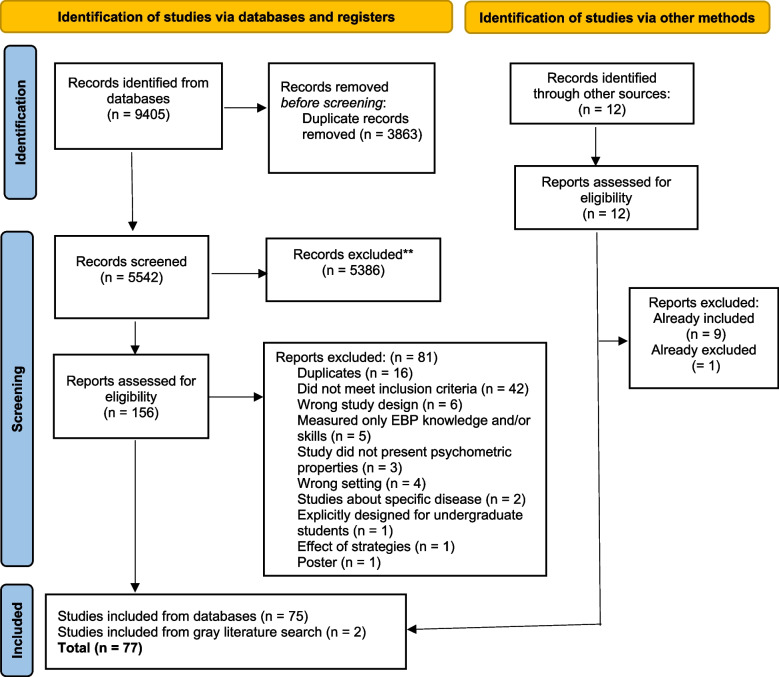
PRISMA flow diagram of the selection process

The search strategy identified 9405 studies. Five thousand five hundred and forty-two studies were screened for eligibility, and 156 were assessed in full text. Seventy-five studies were selected for inclusion. In addition, two studies were included via a search in gray literature. A total of 77 studies were included in the review. The PRISMA flow diagram is presented in Fig. 1.

### Study characteristics

The 77 included studies [36–111] comprised 34 instruments measuring EBP attitudes, behavior or self-efficacy, alone or combined. Twenty-four instruments measured EBP attitudes, 21 measured behavior, and 16 measured EBP self-efficacy. Most instruments were multidimensional and included different subscales (*n* = 25). Eight instruments were unidimensional, and two had indeterminate dimensionality. Nurses were most frequently represented in the included studies (*n* = 53), followed by physiotherapists (*n* = 19), occupational therapists (*n* = 10), medical doctors (*n* = 14), mental health workers (*n* = 16), and social workers (*n* = 7). Ten of the included instruments had been validated in three or more healthcare disciplines [36, 45, 56, 66, 68, 81, 85, 89, 111]. Seven instruments had been validated in two healthcare disciplines [47, 62, 63, 73, 75, 76, 82] and 17 had been validated in only one discipline [48, 64, 65, 71, 78–80, 87, 93, 95, 96, 102, 105, 107, 109, 110]. Details of the included studies and participants are presented in Additional file 3.

### Quality assessment and results of development and content validity studies

Of the 77 studies included, 33 focused on instrument development and 18 focused on content validity on already developed instruments. Table 1 summarizes the quality assessment, rating, and quality of evidence on the development and content validity per instrument.

**Table 1:** Summarized results on quality assessment, rating, and quality of evidence on the development and content validity per instrument

	Development		Content validity (COSMIN box 1 and 2)					
	Concept elicitation (COSMIN box 1a)		Relevance		Comprehensiveness		Comprehensibility	
Instrument (ref)	COSMIN Quality rating	Sample involved?	Rating of results	Quality of evidence	Rating of results	Quality of evidence	Rating of results	Quality of evidence
EBPAS [36, 41, 97–99]	Doubtful	Yes	( +)	Very low*	(–)	Very Low*	( +)	Moderate (rob)
EBPAS-50 [45]	Doubtful	Yes	( +)	Very low *	( +)	Very low *	( +)	Very low *
EBPAS-36 [47, 100]	Doubtful	Yes	( +)	Low (rob)	(–)	Very low *	( +)	Moderate (rob)
EBPQ [48, 49, 51, 52, 54, 101]	Doubtful	Yes	( +)	Very low *	( +)	Very low *	( +)	Low (incon + rob)
EBP Beliefs [56, 58, 59, 99]	Inadequate	No	( +)	Very low *	( +)	Very low *	( +)	Moderate (rob)
EBP Beliefs-Short [102]	Inadequate	No	( +)	Very low *	( +)	Very low *	( +)	Very low *
EBP Implement [56, 58]	Inadequate	No	( +)	Very low *	( +)	Very low *	( +)	Moderate (rob)
EBP Implement-Short [102]	Inadequate	No	( +)	Very low *	( +)	Very low *	( +)	Very low *
Ethiopian EBP Implement [111]	Inadequate	No	( +)	Very low *	( +)	Very low *	( +)	Low (rob × 2)
Al Zoubi Questionnaire [62]	Doubtful	Yes	( +)	Very low *	( +)	Very low *	( +)	Very low *
EBPP-S [63]	Inadequate	No	( ±)	Very low *	(–)	Very low *	--	--
Jette [64, 103]	Inadequate	No	( +)	Very low *	( +)	Moderate (rob)	( +)	Moderate (rob)
Bernhardsson [65]	Inadequate	No	( +)	Very low *	( +)	Low (rob × 2)	( +)	Low (rob × 2)
EBP inventory [66, 67]	Doubtful	Yes	( +)	Low (rob × 2)	( +)	Very low *	( +)	Moderate (rob)
EPIC [68]	Doubtful	No	( +)	Low (rob × 2)	( +)	Low (rob × 2)	( +)	Low (rob × 2)
MPAS [71]	Inadequate	No	( +)	Very low *	(–)	Very low *	( +)	Very low *
EBPPAS [73]	Inadequate	No	( +)	Very low *	( +)	Very low *	( +)	Very low *
SE-EBP [76]	Doubtful	Yes	( +)	Very low *	( +)	Very low *	( +)	Very low *
EBPSE [78]	Doubtful	Yes	( +)	Very low *	( +)	Very low *	( +)	Very low *
EBP Capability Beliefs [79]	Inadequate	No	( +)	Very low *	( +)	Very low *	( +)	Very low *
HEAT [80]	Doubtful	Yes	( +)	Very low *	( +)	Very low *	( +)	Very low *
EBP-KABQ [81]	Doubtful	No	( +)	Very low *	( +)	Very low *	( +)	Very low *
Quick EBP-VIK [82, 84]	Doubtful	No	( +)	Very low *	( +)	Very low *	( +)	Moderate (rob)
HS-EBP [85]	Adequate	Yes	( +)	Very low *	( +)	Very low *	( +)	Very low *
EBPRS [87, 88]	Inadequate	No	( ±)	Very low *	(-)	Very low *	( +)	Very low *
EBP2 [89, 90, 92]	Doubtful	No	( +)	Very low *	( +)	Very low *	( +)	Moderate (rob)
ISP-D [93]	Adequate	Yes	( +)	Low (rob × 2)	( +)	Very low *	( +)	Low (rob × 2)
EBNAQ [95]	Doubtful	Yes	( +)	Low (rob × 2)	( +)	Very low *	( +)	Low (rob × 2)
Diermayr [96]	Doubtful	Yes	( +)	Very low *	(-)	Very low *	( +)	Very low *
EBP-COQ Prof [105, 106]	Doubtful	Yes	( +)	Low (rob × 2)	( +)	Very low *	( +)	Moderate (rob)
EIDM competence measure [107]	Adequate	Yes	( +)	Moderate (rob)	( +)	Very low *	( +)	Moderate (rob)
I-SABE [108]	Doubtful	Yes	( +)	Low (rob × 2)	( +)	Very low *	( +)	Low (rob × 2)
Noor EBM [109]	Doubtful	Yes	( +)	Very low *	( +)	Very low *	( +)	Low (rob × 2)
EBP-CBFRI [110]	Doubtful	No	( +)	Very low *	( +)	Very low *	( +)	Very low *

Overall rating of results: ( +) = sufficient; ( −) = insufficient; (?) = indeterminate; ( ±) = inconsistent

Quality assessment: VG = very good; A = adequate; D = doubtful; I = inadequate

Quality of evidence: Modified GRADE approach [28, 31]. Quality levels: high, moderate, low, and very low

Reasons for downgrade: risk of bias = “RoB”, Inconsistency = “Incon”, Imprecision = “Impre”, Indirectness = “Indir”

“--”: No grade due to lack of questionnaire access

^*^When based only on reviewer’s rating, i.e., not enough evidence from **or** inadequate quality of development study and not enough evidence from **or** inadequate quality of content validity study

The quality of concept elicitation (development of items) was rated as “adequate” in three studies [85, 93, 107], where a clearly reported and appropriate method was used and a sample representing the target population was involved. A further 19 studies received a “doubtful” quality rating [36, 45, 47, 48, 62, 66, 68, 76, 78, 80–82, 89, 95, 96, 105, 108–110]. Some of these studies used qualitative methods to generate items, but the method, or parts of it, was not clearly described. In other studies, it was doubtful whether the included sample was representative of the target population, and some used quantitative methods. Some studies were rated as “doubtful” if it was stated that authors of these studies had talked or discussed the items with relevant healthcare professionals as a part of concept elicitation, but it was doubtful whether this method was suitable. Finally, 12 studies received an “inadequate” quality rating for concept elicitation [56, 63–65, 71, 73, 79, 87, 102, 111]. In these cases, it was clear that no qualitative methods that involved members of the target population were used when generating items. The item generation was usually based on theory, research, or existing instruments.

Content validity was also assessed as part of the development studies with cognitive interviews or pilot tests or in separate content validity studies performed after the instrument was developed, primarily studies translating an instrument. Some development studies assessed comprehensibility [47, 56, 65, 68, 73, 76, 78, 82, 87, 89, 93, 95, 105, 107–111] or comprehensiveness [65, 68] with interviews or pilot tests on samples representing the target population. These were rated as either “adequate” [93, 107] or “doubtful” quality [47, 56, 65, 68, 73, 76, 78, 82, 87, 89, 95, 105, 108–111]. The rest of the development studies could not be rated, either because it was unclear whether a pilot test or interview was performed, or which aspect of content validity was assessed. Most of the content validity studies assessed comprehensibility [41, 49, 51, 52, 54, 58, 59, 84, 88, 90, 92, 97–101, 103, 106] and only a few assessed relevance or comprehensiveness [59, 84, 88, 99, 103]. All content validity studies were rated as doubtful quality [41, 49, 51, 52, 54, 58, 59, 84, 88, 90, 92, 97–101, 103, 106].

### Results of synthesis and certainty of evidence on content validity

With the combined results from each study's ratings of relevance, comprehensiveness, and comprehensibility and the reviewers’ ratings, each instrument was given an overall rating (Table 1). Most instruments were rated as sufficient ( +) on relevance and comprehensibility, and only 6 out of 34 instruments were rated as insufficient ( −) on comprehensiveness. The quality of evidence was graded as “very low” in most cases, primarily due to no content validity studies (or inadequate quality) and not enough evidence from (or inadequate quality of) the development studies. The overall grade was, in these cases, based solely on the reviewers’ ratings and was therefore downgraded to “very low” [26].

Seven instruments (EBPAS-36, EBP Inventory, EPIC, ISP-D, EBNAQ, EBP-COQ Prof, and I-SABE) had “low” quality evidence of sufficient “relevance” from concept elicitation studies of doubtful quality [26]. One instrument (EIDM competence measure) had “moderate” quality evidence of sufficient “relevance” from a development study of adequate quality. Two instruments (EPIC and Bernhardsson) had “low”, and another (Jette) had “moderate” quality evidence of sufficient “comprehensiveness” from a development study of doubtful quality and a content validity study of doubtful quality [26].

Ten instruments (EBPAS, EBPAS 36, EBP inventory, EBP Beliefs, EBP Implement, Jette, Quick EBP VIK, EBP2, EBP-COQ Prof, and EIDM competence measure) had “moderate” quality evidence of sufficient “comprehensibility” from content validity studies of doubtful quality or development studies of adequate quality [26]. In addition, eight instruments (EBPQ, EPIC, Bernhardsson, ISP-D, EBNAQ, I-SABE, Noor EBM, and Ethiopian EBP Implement) had “low” quality evidence of sufficient “comprehensibility” from development studies of doubtful quality or content validity studies of doubtful quality but with inconsistent results [26].

### Quality assessment and results of structural validity and internal consistency studies

Structural validity was assessed in 63 studies and internal consistency in 69 studies. The quality assessment and results of rating of structural validity and internal consistency per study are presented in detail in Additional file 4.

To test structural validity, most studies used exploratory factor analyses (EFA) (*n* = 26) or confirmatory factor analyses (CFA) (*n* = 34), and two studies used IRT/Rasch analyses. Since CFA is preferred over EFA in the COSMIN methodology [31], only the results of CFA were rated in studies where both EFA and CFA were conducted. The quality of structural validity testing was rated as “very good” in 33 studies [36–38, 40, 42–44, 47, 49, 50, 53, 55, 72, 74, 75, 77, 79–81, 84, 86, 88, 90, 92, 94, 97–100, 105, 106, 110], “adequate” in 19 studies [39, 45, 48, 51, 52, 57, 58, 60, 62, 69, 76, 89, 91, 95, 108, 109, 111], “doubtful” in 9 studies [46, 56, 59, 61, 63, 83, 102], and as “inadequate” in two studies [66, 73]. In both cases inadequate ratings were given due to low sample sizes [31].

To test internal consistency of the items, most studies calculated and reported a Cronbach’s alpha (*n* = 67), and two studies calculated and reported a person separation index. The quality of internal consistency calculations was rated as “very good” in 64 studies [36–39, 41–45, 47–63, 66, 67, 69, 71–81, 83, 84, 86, 88–92, 94, 95, 97, 99–102, 104–106, 108, 110] and as “inadequate” in five studies [46, 60, 98, 109, 111]. Inadequate ratings were given when a Cronbach’s alpha was not reported for each unidimensional subscale in a multidimensional instrument [31].

### Results of synthesis and certainty of evidence of structural validity and internal consistency

Qualitatively summarized results, overall rating, and quality of evidence (COSMIN GRADE) on structural validity and internal consistency per instrument are presented in detail in Tables 2 and 3.

**Table 2:** Qualitatively summarized results, overall rating, and quality of evidence (GRADE) on *structural validity* per instrument

Instrument (ref)	Summarized result on structural validity	Rating	Quality of evidence	
			Quality level	Reason
EBPAS [36–40, 42–44, 97–99]	EFA and CFA. Four factors/subscales. Inconsistent results. Results for seven studies met the criteria (SRMR < 0.08), three studies did not (SRMR > 0.08/RMSEA > 0.06)	( +)	Moderate	− 1 incon
EBPAS-50 [45, 46]	EFA and CFA. Eight new factors. Inconsistent results, different number of factors in two studies. EFA criteria were met on eight-factor structure, no fit indices reported on CFA	( +)	Low	− 1 incon, − 1 RoB
EBPAS-36 [47, 100]	CFA showed a 36-item 12-factor scale. Inconsistent results. One study met the criteria (RMSEA = 0.052) and one study did not (RMSEA = 0.64)	( ±)	No grade	
EBPQ [48–53, 55]	EFA and CFA. Three factors/subscales. Inconsistent results. Results for five studies met the criteria for CFA (SRMR < 0.08) or criteria for EFA, two studies did not	( +)	Moderate	− 1 incon
EBP Beliefs [56–60]	EFA. Disagreement about dimensionality between five studies. Results summarized in subgroups (unidimensional/multifactorial) 1. Unidimensional (single factor): criteria for EFA were met in both (two) 2. Multifactorial (four factors): inconsistent results. Criteria not met in two studies due to cross-loading, and the third for not reporting cross-loading	1: ( +) 2: ( −)	Moderate Moderate	− 1 RoB − 1 incon
EBP Beliefs- Short [102]	EFA. One factor. Factor loading > 0.70, Eigenvalue = 2.25	( +)	Low	− 2 RoB
EBP Implement [56, 58, 60, 61]	EFA. Disagreement about dimensionality between four studies. Results are summarized in subgroups (unidimensional/multifactorial) 1. Unidimensional (single factor): criteria for EFA were met in one study 2. Multifactorial (four/five/two factors): inconsistent results. Criteria not met in two studies due to cross-loading, and one study rated as indeterminate due to not reporting eigenvalue, total variance explained, or cross-loading	1: ( +) 2: ( −)	Low Moderate	− 2 RoB − 1 incon
EBP Implement-Short [102]	EFA. One factor. Factor loading > 0.85, Eigenvalue = 2.46	( +)	Low	− 2 RoB
Ethiopian EBP Implement [111]	EFA (two factors): Factor loadings > 0.40, Eigenvalues > 1, cross-loading, no cross loadings	( +)	Moderate	− 1 Rob
Al Zoubi Questionnaire [62]	IRT/Rasch: Unidimensionality: CFI, TLI, RMSEA or SRMR not reported. Local independence: items correlating > 0.3 led to removal. Monotonicity: not reported. Model fit (× 2 test): × 2 > 0.01 on three out of four subscales	(?)	No grade	
EBPP-S [63]	CFA confirmed a three-factor scale. Model fit: CFI = .96, and RMSEA = .06	( +)	Low	− 2 RoB
EBP inventory [66]	EFA. Four factors/subscales. Factor loadings > 0.30, cross-loading, tot variance explained, and eigenvalue not reported	(?)	No grade	
EPIC [69]	Unidimensional scale. EFA: all items loaded into one single factor > 0.4, tot explained variance = 71%, < 10% cross-loading	( +)	Moderate	− 1 RoB
MPAS [72]	CFA. Unidimensional model, and a modified five-item model had the best fit: US: CFA: RMSEA = 0.030, CFI 0.998 Korea: CFA: RMSEA = < 0.05, CFI > 0.95	( +) ( +)	High High	
EBPPAS [73, 74]	EFA and CFA. Inconsistent result. One study met the criteria for EFA (factor loading > 0.3, eigenvalues > 1, and < 10% cross-loading). The other study did not meet the criteria for CFA (CFI = 0.90)	( ±)	No grade	
EBPPAS-s [75]	CFA. Revised four-factor model, 37 items. Model fit: CFI = 0.90, RMSEA = 0.06	( −)	High	
SE-EBP [76, 77]	EFA and CFA. Three factors/subscales. Inconsistent results. One study met the criteria for EFA (factor loading > 0.3, tot explained variance = 73.01%, and < 10% cross-loading). One study did not meet the criteria for CFA (CFI = 0.91)	( ±)	No grade	
EBP Capability Beliefs [79]	IRT/Rasch: Unidimensionality: CFI, TLI, RMSEA or SRMR not reported. Local independence: two small cross-loadings on the first factor; 0.23 and 0.30. Monotonicity: not reported. Model fit (× 2 test): × 2 = 42, 71	(?)	No grade	
HEAT [80]	EFA and CFA. Four-factor model. CFA model fit: SRMR = 0.063	( +)	High	
EBP-KABQ [81]	CFA: Four-factor modified model. Model fit: CFI = 0.89	( −)	High	
Quick EBP-VIK [83, 84]	EFA and CFA. Three-factor model, inconsistent results. Model fit: CFI = 0.957	( +)	Moderate	− 1 Incon
HS-EBP [86]	EFA and CFA. Five factors. Model fit: CFI = 0.99, RMSEA = 0.047, SRMR = 0.067	( +)	High	
EBPRS [88]	EFA and CFA. Four factors. Model fit: CFI = 0.96, TLI = 0.96, SRMR = 0.058	( +)	High	
EBP2 [89–92]	EFA and CFA. Inconsistent results. Five factors in three out of four studies. One study met the criteria for EFA (factor loading > 0.3, tot explained variance = 63%, and < 10% cross-loading), one did not report enough information to be rated, the third did not meet criteria for CFA (CFI = 0.69). Overall rating is inconsistent	( ±)	No grade	
ISP-D [94]	CFA. Four-factor model. Model fit: SRMR = 0.075	( +)	High	
EBNAQ [95]	PCA (EFA) with Promax rotation showed three factors explaining a total variance of 54.7%, factor loadings > 0.30 and cross-loading < 10%	( +)	Moderate	− 1 RoB
EBP-COQ Prof [105, 106]	CFA. Four-factor model. Model fit: CFI = 0.93 and 0.82 in two studies	( −)	High	
I-SABE [108]	EFA. Four factors. Tot variance explained = 52.6%, factor loading > 0.3, cross-loading > 10%	( −)	Moderate	− 1 RoB
Noor EBM [109]	EFA. 1. Attitude scale (five factors): factor loading = > 0.4, tot variance = 66.3%, cross-loading not reported. 2. Practice scale (two factors): factor loading = > 0.4, tot variance = 55.4%, cross-loading not reported	1: (?) 2: (?)	1,2: no grade	
EBP-CBFRI [110]	CFA. Five-factor model. Model fit: RMSEA = 0.05	( +)	High	

Overall rating of results: ( +) = sufficient result; ( −) = insufficient result; (?) = indeterminate result; ( ±) = inconsistent results

Quality of evidence: Modified GRADE-approach [28, 31]. Quality levels: high, moderate, low, and very low

Reasons for downgrade: Risk of bias = “RoB”, Inconsistency = “Incon”, Imprecision = “Impre”, Indirectness = “Indir”

*CFI* Comparative fit index, *RMSEA* Root mean square error of approximation, *SRMR* Standardized square residual, *EFA* Exploratory factor analysis, *CFA* Confirmatory factor analysis

**Table 3:** Qualitatively summarized results, overall rating, and quality of evidence (GRADE) on *internal consistency* per instrument

Instrument (reference)	Summarized result on internal consistency (overall rating)	Rating	Quality of evidence	
			Quality level	Reason
EBPAS [36–39, 41–44, 97, 99]	Cronbach’s alpha range per subscale: 1. Requirements 0.88–0.94, 2. Appeal 0.72–0.80, 3. Openness 0.73–0.84, 4. Divergence 0.51–0.85	1–3: ( +) 4: ( −)	Moderate Moderate	− 1 struc val − 1 struc val
EBPAS-50 [45, 46]	Cronbach’s alpha ranged from 0.77 to 0.92 on the eight new subscales	( +)	Low	− 2 struc val
EBPAS-36 [47, 100]	Cronbach’s alphas range per subscale: 1. Appeal, Fit, Balance and Divergence = 0.61–0.69, 0.62–0.68, 0.64–0.65, and 0.66–0.68. 2. All other subscales > 0.70. Indeterminate rating (?) since criteria for sufficient structural validity not met	(?)	No grade	
EBPQ [48–55, 101]	Cronbach’s alpha range per subscale: 1. Attitudes: 0.68–0.83, 2. Practice: 0.74–0.93, 3. Knowledge/skills: 0.90–0.96	1: ( +) 2,3: ( +)	Low Moderate	− 1 struc val, − 1 incon − 1 struc val
EBP Beliefs [56–60]	Disagreement about dimensionality between give studies. Results are summarized in subgroups (unidimensional/multifactorial) 1. Unidimensional (single factor): Cronbach’s alpha range = 0.86–0.90 2. Multifactorial (four factors): Indeterminate rating (?) since criteria for sufficient structural validity not met	1: ( +) 2: (?)	Moderate No grade	− 1 struc val
EBP Beliefs- Short [102]	Cronbach’s alpha on scale = 0.89	( +)	Low	− 2 Struc val
EBP Implement [56, 58, 60, 61]	Disagreement about dimensionality between four studies. Results are summarized in subgroups (unidimensional/multifactorial) 1. Unidimensional (single factor): Cronbach’s alpha = 0.96 2. Multifactorial (four/five/two factors): Indeterminate rating (?) since criteria for sufficient structural validity not met	1: ( +) 2: (?)	Low No grade	− 2 struc val
EBP Implement- Short [102]	Cronbach’s alpha on scale = 0.81	( +)	Low	− 2 Struc val
Ethiopian EBP Implement [111]	Cronbach’s alpha not reported for each subscale. total scale = 0.83	(?)	No grade	
Al Zoubi Questionnaire [62]	Person separation index per subscale: Attitudes = 0.63, Self-efficacy = 0.80, Knowledge = 0.81, Resources = 0.86. Indeterminate rating (?) since criteria for sufficient structural validity not met	(?)	No grade	
EBPP-S [63]	Cronbach’s alpha per subscale: 1. Attitudes = 0.89, 2. Knowledge = 0.90, 3. Behavior = 0.83	1–3 = ( +)	Low	− 2 struc val
EBP inventory [66, 67]	Cronbach’s alpha range per subscale: 1. Decision making = 0.60–0.64**,** 2. All other subscales > 0.70. Indeterminate rating (?) since criteria for sufficient structural validity not met	(?)	No grade	
EPIC [69]	Cronbach’s alpha on scale: 0.89	( +)	Low	− 1 Struc val, − 1 RoB
MPAS [71, 72]	Cronbach’s alpha range per sample: 1. USA = 0.78–0.80, 2. Korea: 0.65	1: ( +) 2: ( −)	High Low	− 1 RoB
EBPPAS [73, 74]	Cronbach’s alpha range per subscale: Familiarity = 0.91–0.92, Attitudes = 0.83–0.90, Feasibility = 0.57–0.63, Intentions = 0.80–0.86, Currently engaged = 0.86–0.87. Indeterminate rating (?) since criteria for sufficient structural validity not met	(?)	No grade	
EBPPAS-s [75]	Cronbach’s alpha per subscale: Familiarity = 0.93, Attitudes = 0.92, Feasibility = 0.74, Currently engaged = 0.91. Indeterminate rating (?) since criteria for sufficient structural validity not met	(?)	No grade	
SE-EBP [76, 77]	Cronbach’s alpha range per subscale: identifying the clinical problem = 0.89–0.91, searching for evidence = 0.90–0.96, implementing evidence into practice = 0.93–0.96. Indeterminate rating (?) since criteria for sufficient structural validity not met	(?)	No grade	
EBPSE [78]	Cronbach’s alpha = 0.95. Indeterminate rating (?) since no evidence of unidimensionality	(?)	No grade	
EBP Capability Beliefs [79]	Person separation index = 0.92. Indeterminate rating (?) since no evidence of unidimensionality	(?)	No grade	
HEAT [80]	Cronbach’s alpha per subscale: EBP frequency = 0.82, EBP ability = 0.89, EBP desire = 0.92, EBP barriers = 0.80	( +)	High	
EBP-KABQ [81]	Cronbach’s alpha per subscale: Knowledge = 0.66, Attitude = 0.75, Behavior = 0.77, Outcome = 0.83. Indeterminate rating (?) since criteria for sufficient structural validity not met	(?)	No grade	
Quick EBP-VIK [83, 84]	Cronbach’s alpha range per subscale: 1. Value = 0.78–0.89, 2. Knowledge = 0.92–0.93, 3. Implement = 0.66–0.91	1–2: ( +) 3: ( ±)	Moderate No grade	− 2: Struc val
HS-EBP [86]	Cronbach’s alpha per subscale: Beliefs and attitudes = 0.93, Results from scientific research = 0.96, Development of professional practice = 0.84, Assessment of results = 0.94, Barriers or Facilitators = 0.91	( +)	High	
EBPRS [88]	Cronbach’s alpha per subscale: Informational needs: 0.85, EBP knowledge: 0.78, EBP attitude: 0.87, Workplace culture: 0.84	( +)	High	
EBP2 [89–92, 104]	Cronbach’s alpha range per subscale (five-factor model): Relevance: 0.91–0.94, Sympathy: 0.66–0.80, Terminology: 0.94–0.98, Practice: 0.85–0.923, Confidence: 0.93–0.94. Indeterminate (?) rating since criteria for sufficient structural validity not met	(?)	No grade	
ISP-D [94]	Cronbach’s alpha per subscale (four-factor model): 1. Attitudes = 0.75, 2. Subjective norms = 0.72, 3. Perceived behavioral control = 0.63, 4. Behavioral intention = 0.84	1, 2, 4: ( +) 3: ( −)	1, 2, 4: High 3: High	
EBNAQ [95]	Cronbach’s alpha per subscale (three-factor model): 1. Beliefs and expectations = 0.86, 2. Intention of conduct = 0.63, 3. Feelings toward the EBN = 0.70	1, 3: ( +) 2: ( −)	Moderate Moderate	− 1 struc val − 1 struc val
EBP-COQ Prof [105, 106]	Cronbach’s alpha per subscale: 1. Attitude toward EBP = 0.888, 2. EBP knowledge = 0.948, 3. EBP skills = 0.817, 4. EBP utilization = 0.840	(?)	No grade	
I-SABE [108]	Cronbach’s alpha per subscale: 1. EBP self-efficacy = 0.76, 2. Behavior = 0.30, 3. Attitudes = 0.64, 4. Results = 0.84. Indeterminate (?) rating since criteria for sufficient structural validity not met	(?)	No grade	
Noor EBM [109]	Cronbach’s alpha on total scales (not per subscale): 1.Attitude scale = 0.81, 2. Practice scale = 0.84. Indeterminate (?) rating since criteria for sufficient structural validity not met	(?)	No grade	
EBP-CBFRI [110]	Cronbach’s alpha range on subscales = 0.92–0.95	( +)	High	

Overall rating of results: ( +) = sufficient result; ( −) = insufficient result; (?) = indeterminate result; ( ±) = inconsistent results

Quality of evidence: modified GRADE approach [28, 31]. Quality levels: high, moderate, low, and very low

Reasons for downgrade: risk of bias = “RoB”, Inconsistency = “Incon”, imprecision = “Impre”, indirectness = “Indir”

α = Cronbach’s alpha

Eighteen instruments were rated overall as sufficient ( +) structural validity (EBPAS, EBPAS-50, EBPQ, EBP Belief-single factor, EBP Implement-single factor, EBPP-S, EPIC, MPAS, HEAT, Quick EBP-VIK, HS-EBP, EBPRS, ISP-D, EBNAQ, EBP Beliefs short, EBP Implement Short, EBP-CBFRI, and Ethiopian EBP Implement), with the quality of evidence ranging from “high” to “low.” Reasons for downgrading the quality of evidence were either “risk of bias” or “inconsistency”. Six instruments were rated overall as insufficient ( −) structural validity (EBP belief-multifactorial, EBP implement-multifactorial, EBPPAS-s, EBP-KABQ, EBP-COQ Prof, and I-SABE), with the quality of evidence ranging from “high” to “moderate.” The reasons for downgrading were “inconsistency” and “risk of bias.” Four instruments were rated overall as inconsistent (+ / −) structural validity (EBPPAS, SE-EBP, EBP2, and EBPAS-36). In these three cases, results were inconsistent and it was not possible to give an overall rating as sufficient or insufficient (e.g., an overall rating based on the majority of studies) [31]. Finally, four instruments were rated overall as indeterminate (?) structural validity (Al Zoubi Q, EBP Inventory, EBP capability beliefs, and Noor EBM) because not all the information needed for a sufficient rating was reported [31].

Regarding internal consistency, 16 instruments were rated overall as indeterminate (?) (EBP belief-multifactorial, EBP implement-multifactorial, Al Zoubi Q, EBP Inventory, EBPPAS, EBPPAS-s, SE-EBP, EBPSE, EBP capability beliefs, EBP-KABQ, EBP2, EBP-COQ Prof, I-SABE, Noor EBM, Ethiopian EBP Implement, and EBPAS-36). Most of these instruments had Cronbach’s alpha values that met the criteria for sufficient internal consistency (α > 0.70). However, since evidence of structural validity is a prerequisite of internal consistency, they were rated as indeterminate (?) according to the COSMIN methodology [28]. Furthermore, the summarized result of internal consistency was rated and graded per subscale in cases of multifactorial instruments. This led to several instruments receiving different ratings on different subscales, such as sufficient ( +), insufficient ( −) or inconsistent (+ / −) (EBPAS, MPAS, Quick EBP VIK, ISP-D, and EBNAQ). Seven multifactorial and five unidimensional instruments were rated as sufficient ( +) on all subscales or full scales (EBPAS-50, EBPQ, EBP Beliefs-single factor, EBP Implement-single factor, EBPP-S, EPIC, HEAT, HS-EBP, EBPRS, EBP Beliefs-Short, EBP Implement-Short, and EBP-CBFRI). The quality of evidence ranged from “high” to “low,” and the most common reason for downgrading was that the quality of evidence of structural validity on the same instrument set the starting point for the grading of internal consistency [31].

### Quality assessment and results of reliability and measurement error studies

Reliability was assessed in 22 studies, and measurement error in five studies. The quality assessment and results of the rating of reliability and measurement error per study are presented in detail in Additional file 4.

To test reliability, 18 studies calculated and reported an intraclass correlation coefficient (ICC), two used Pearson’s correlation, and two used the percentage of agreement. The quality of reliability testing was rated as “very good” in two studies [41, 67], “adequate” in 12 studies [39, 64, 66, 69, 83, 84, 89–92, 105, 106], “doubtful” in six studies [46, 50, 52, 54, 70, 96], and as “inadequate” in two studies [65, 103]. Reasons for a “doubtful” rating were that time intervals between measurements were longer than recommended or it was unclear whether respondents were stable between measurements or whether only Pearson’s or Spearman’s correlation coefficients were calculated [31]. The reason for the “inadequate” rating was that no ICC, Pearson’s or Spearman’s correlation coefficients were calculated [31].

To test measurement error, all studies calculated standard error of measurement (SEM), smallest (minimal) detectable change (SDC) or limits of agreement (LoA). Only one study reported information on minimal important change (MIC). The quality of measurement error testing was rated as “very good” in two studies [41, 67], “adequate” in two studies [69, 92], and as “doubtful” in one study [70]. The reason for the “doubtful” rating was that a time interval between measurements was longer than recommended.

### Results of synthesis and certainty of evidence of reliability and measurement error

Qualitatively summarized results, overall rating, and quality of evidence (COSMIN GRADE) on reliability and measurement error are presented in detail in Tables 4 and 5.

**Table 4:** Qualitatively summarized results, overall rating, and quality of evidence (GRADE) on *reliability* per instrument

Instrument (reference(s))	Summarized result on reliability	Overall rating	Quality of evidence	
			Quality level	Reason
EBPAS [39, 41]	ICC range per subscale: 1. Requirements 0.55–0.80, 2. Appeal 0.40–0.56, 3. Openness 0.64–0.71, 4. Divergence 0.48–0.74	1, 3, 4: ( ±) 2: ( −)	No grade High	
EBPAS-50 [46]	Pearson’s correlation total scale = 0.344. Indeterminate rating (?) since ICC not calculated	(?)	No grade	
EBPQ [50, 52, 54]	ICC range per subscale: 1. Attitudes: 0.44–0.86, 2. Practice: 0.78–0.84, 3. Knowledge/skills: 0.70–0.86	1: ( ±) 2, 3: ( +)	No grade Moderate	− 1 RoB
Jette [64, 103]	ICC ranged from 0.37 to 0.90 (50% > 0.70). Indeterminate rating (?) since ICC per subscale not reported	(?)	No grade	
Bernhardsson [65]	Indeterminate rating (?) since no ICC or Pearson’s or Spearman’s correlations reported	(?)	No grade	
EBP inventory [66, 67]	ICC range per subscale: 1. Decision making = 0.71–0.78, 2. Subjective norm = 0.63–0.86, 3. Attitude = 0.52–0.82, 4. Perceived behavioral control = 0.80–0.83, 5. Intention and behavior = 0.76–0.86	1, 4, 5: ( +) 2, 3: ( +)	High Moderate	− 1 Incon
EPIC [69, 70]	ICC range = 0.89–0.92	( +)	Moderate	− 1 RoB
Quick EBP-VIK [83, 84]	ICC range per subscale: 1. Value = 0.51–0.57, 2. Knowledge = 0.70–0.88, 3. Implement = 0.63–0.84	1: ( −) 2: ( +) 3: ( ±)	High High No grade	
EBP2 [89–92]	ICC range per subscale (five-factor model). 1. Relevance = 0.69–0.94, 2. Terminology = 0.79–0.94, 3. Confidence = 0.76–0.95, 4. Practice = 0.45–0.92, 5. Sympathy = 0.47–0.77	1, 4: ( +) 2, 3: ( +) 5: ( ±)	Moderate High No grade	Incon
EBP Diermayr [96]	Mean ICC 0.67 (range 0.40–0.89). Indeterminate rating (?) since ICC per subscale not reported	(?)	No grade	
EBP-COQ Prof [105, 106]	ICC per subscale (four-factor model): 1. Attitude toward EBP = 0.840, 2. EBP knowledge = 0.966, 3. EBP skills = 0.815, 4. EBP utilization = 0.876	( +)	Moderate	− 1 impre

*ICC* = Intraclass correlation coefficient

Rating of results: ( +) = sufficient result; ( −) = insufficient result; (?) = indeterminate result; ( ±) = inconsistent results

Quality of evidence: modified GRADE approach [28, 31]. Quality levels: high, moderate, low, and very low

Reasons for downgrade: risk of bias = “RoB”, inconsistency = “Incon”, imprecision = “Impre”, indirectness = “Indir”

**Table 5:** Qualitatively summarized results, overall rating, and quality of evidence (GRADE) on *measurement error* per instrument

Instrument (reference)	Summarized result on measurement error	Overall rating	Quality of evidence	
			Quality level	Reason
EBPAS [41]	LoA < SRD on all subscales	**( +)**	Moderate	− 1 impre
EBP inventory [67]	MDC95 (absolute value) range from 3.5 to 7.2. Indeterminate rating (?) since MIC not defined	**(?)**	No grade	
EPIC [69, 70]	MDC95 (absolute value) range from 4.1 to 4.6. Indeterminate rating (?) since MIC not defined	**(?)**	No grade	
EBP2 [92]	SEM: ranging from 0.29 to 0.44. Indeterminate rating (?) since MIC not defined	**(?)**	No grade	

*SEM* Standard error of measurement, *LoA* Limits of agreement, *SDC* Smallest detectable change, *MIC* Minimal important change

Overall rating of result: ( +) = sufficient result; ( −) = insufficient result; (?) = indeterminate result; ( ±) = inconsistent results

Quality of evidence: modified GRADE approach [28, 31]. Quality levels: high, moderate, low, and very low

Reasons for downgrade: risk of bias = “RoB”, inconsistency = “Incon”, imprecision = “Impre”, indirectness = “Indir”

The summarized result of reliability was rated and graded per subscale in cases of multifactorial instruments. This led to four instruments receiving different overall ratings on different subscales, such as sufficient ( +), insufficient (-) or inconsistent (+ / −) reliability (EBPAS, EBPQ, Quick EBP-VIK, and EBP2). Three instruments were rated overall as sufficient ( +) reliability (EBP inventory, EPIC, and EBP-COQ Prof). The quality of evidence ranged from “high” to “low.” Reasons for downgrading the quality of evidence were either “inconsistency,” “risk of bias” or “imprecision.” Four instruments were rated overall as indeterminate (?) reliability (EBPAS-50, EBP (Jette), EBP (Bernhardsson), and EBP (Diermayr)). The reasons for indeterminate ratings were that ICC was not calculated, not reported or not reported in sufficient detail to allow rating and grading [31].

Regarding measurement error, one instrument was rated overall as sufficient ( +), with the quality of evidence graded as “moderate.” It was downgraded for imprecision due to the small sample size. Since MIC was not defined, three other instruments were rated overall as indeterminate (?) measurement error [31].

## Discussion

This review sought to summarize measurement properties of existing instruments that measure healthcare professionals’ EBP attitudes, behaviors, and self-efficacy. We evaluated the instruments’ development process, content validity, structural validity, internal consistency, reliability, and measurement error. Thirty-four instruments measuring EBP attitudes, behavior or self-efficacy, alone or combined, were identified.

The assessment of instrument development studies revealed that only three instruments received an “adequate” quality rating on concept elicitation (HS-EBP, ISP-D, and EIDM competence measure) [85, 93, 107]. The rest were rated “doubtful” or “inadequate.” Reasons for “doubtful” ratings were mainly related to the quality of the qualitative methods used to generate items and “inadequate” ratings were given when no qualitative methods seemed to have been used. The use of well-designed qualitative methods when constructing the items is emphasized in the updated COSMIN methodology (2018) that was used in this review [26]. However, over two-thirds of the development studies included in this review were published before the updated COSMIN methodology was published in 2018 [26]. Thus, assessing instrument development studies based on a detailed and standardized methodology to which the developers did not have access when developing instruments can be somewhat strict. At the same time, the quality of the development process (concept elicitation) has not, to our knowledge, been rated in detail in previous reviews of EBP instruments [20–25]. Thus, our findings underpin the importance that future instrument development studies should involve the target population using qualitative methods to generate items for an EBP instrument.

The summarized results on internal consistency showed that several instruments were rated overall as indeterminate (?) despite meeting the criteria for a sufficient ( +) rating (Cronbach’s alpha > 0.70). Although measuring “how well items correlate,” Cronbach’s alpha is often misinterpreted as a measure of the dimensionality of a scale. Whether the scores on a scale reflect the dimensionality of the construct measured is defined as structural validity and is most often assessed by factor analysis ([112], p. 169–170, [113]). Evidence of unidimensionality of a scale or subscale is an assumption that needs to be verified before calculating Cronbach’s alpha to assess the interrelatedness of the items [113]. Though internal consistency helps assess whether items on a scale or subscale are related, evidence of structural validity must come first to ensure that the interrelated items are on a scale or subscale that also reflects the construct's dimensionality. The rating of internal consistency in this review is based on the COSMIN criteria for whether or not evidence of unidimensionality on the scale exists [31]. Indeterminate (?) ratings on internal consistency alone will not lead to an instrument not being recommended in this review, since this requires high-quality evidence of insufficient (–) measurement properties.

This review’s target population was healthcare professionals, and the number of healthcare disciplines on which an instrument was validated was one of the factors considered when making categories of recommendations. While 17 out of the 34 included instruments were validated on two or more healthcare disciplines, 17 were validated on only one [48, 64, 65, 71, 78–80, 87, 93, 95, 96, 102, 105, 107, 109, 110]. When an instrument is validated in only one healthcare discipline, the results from a validation study may not apply if an instrument is used on a population that differs from the one on which the instrument was validated ([114], p. 230–231). Studies have shown that there may be differences between healthcare disciplines in terms of self-reported levels of EBP knowledge, attitudes, and behavior [115, 116]. It is unknown whether interdisciplinary differences in EBP knowledge, attitudes or behavior would directly affect how the items in a questionnaire are understood or to what degree they are perceived as relevant. However, knowing that a questionnaire only can be considered valid for the population on which it has been validated ([112], p.58–59), readers of this review should bear in mind that the results may not be generalizable to other populations. Readers should have a clear conception of the population on which the instrument is tested and of the population intended to target when choosing an instrument for use in future studies or clinical practice. This review’s inclusion of studies from various healthcare disciplines may have contributed new knowledge to the current evidence base, identifying several valid instruments over at least two disciplines.

Most of the instruments included in this review were initially developed in English and in different English-speaking countries. Several of these instruments have been translated into other languages and used in various countries. Ideally, an instrument translation process should be conducted according to well-known guidelines to ensure that a translated instrument is valid in another language [112, 117, 118]. In this review, we did not assess the quality of the translation process, as this was not part of the COSMIN methodology recommendations used to conduct this review [26, 31]. As such, readers are advised to consider the quality of the translation process if they consider using results from studies included in this review that involved translations of instruments.

### Limitations

Variations in definitions of EBP constructs between the included studies presented a challenge in the review process. Clearly defined constructs are essential to instrument development and are a prerequisite for using quantitative questionnaires to measure non-observable constructs like EBP attitudes, self-efficacy, and behavior ([112], p. 151–152). In some cases, the differences in definitions of constructs and use of terminology made it challenging to classify the included instruments in terms of the EBP constructs measured. To meet this challenge, we classified the instruments using the CREATE framework’s definitions of EBP attitudes, self-efficacy, and behavior mentioned earlier in this review [10]. For some instruments, the constructs were defined with names and terminology other than those used in the CREATE framework. The differences in definitions of constructs and use of terminology may also have affected the study selection of this review, with potentially relevant studies being overlooked and not being included. To meet this challenge, all titles and abstracts were screened by two independent review members, and a third reviewer was consulted in cases of uncertainty. Still, relevant studies and instruments may have been missed. Even though EBP theory, models, and frameworks exist, there is still a need to develop a more cohesive and clear theoretical articulation regarding EBP and the measurement of it [10, 119].

Furthermore, all the included instruments are self-reported, the most common method to measure EBP constructs. Some consider only objectively measured EBP outcomes as high-quality instruments due to the potential of recall and social desirability biases in self-reported instruments [16, 17, 22, 23]. Despite the risk of bias, others recommend using self-reported instruments as a practical option when time is an issue and an extensive, objective measurement is practically impossible [119]. In addition, it has been questioned whether the extensive focus on objectivity in EBP instruments is the only right way forward, and qualitative and mixed methods have been suggested for a richer understanding of EBP [119]. The use of a standardized and rigorous methodology (COSMIN) throughout this review may have reduced possible methodological limitations and increased the likelihood that the results and recommendations could be trusted, despite the potential risk of bias connected to self-reported instruments.

### Rationale for recommendations and implications of future research

Recommendations of instruments in this review are based on the summarized results and grading of the evidence concerning the construct and population of interest. The recommendations are guided by the COSMIN methodology but are not categorized similarly [31]. The three categories are categorized based on the number of healthcare disciplines on which the instrument is validated and on the number of EBP constructs the instrument measures. Common for all three categories is that, for an instrument to be recommended, there must be evidence of sufficient ( +) content validity (any level) and no high-quality evidence of any insufficient ( −) measurement properties [31]. Being recommended means that an instrument has the potential to be recommended, even though it does not have exclusively high-quality evidence of sufficient measurement properties. This aligns with research that suggests building upon existing instruments when measuring EBP attitudes, self-efficacy, and behavior [10]. Using and adapting existing instruments could also help to avoid the so-called “one-time use phenomenon,” where an instrument is developed for a specific situation and not further tested and validated in other studies ([120], p.238).

### Recommendations

Instruments validated in at least two healthcare disciplines that measure two or more of the constructs in question (attitudes, behavior, self-efficacy) include the following: EBP Inventory [66], Al Zoubi questionnaire [62], EBPPAS [73], HS-EBP [85], EBP2 [89], and I-SABE [108]. Furthermore, instruments validated in at least two healthcare disciplines but that measure only one of the constructs in question include the following: EBPAS-50 [45], EBP Beliefs (single factor) [56], EBP implement (single factor) [56], EPIC [68], SE-EBP [76], and Ethiopian EBP Implement [111]. Finally, instruments validated in only one discipline that measures one or more of the constructs in question include the following: EBPQ [48], EBP (Jette) [64], EBP (Bernhardsson) [65], EBPSE [78], EBP Capability beliefs [79], HEAT [80], Quick EBP-VIK [82], ISP-D [93], EBNAQ [95], EBP Implement short [102], EIDM competence measure [107], Noor EBM [109], and EBP-CBFRI [110].

## Conclusions

This review identified 34 instruments that measure healthcare professionals’ EBP attitudes, behaviors, or self-efficacy. Seventeen instruments were validated in two or more healthcare disciplines. Despite the varying quality of instrument development and content validity studies, most instruments received sufficient ( +) ratings on content validity, though with a “very low” quality of evidence. The overall rating of structural validity, internal consistency, reliability, and measurement error varied, as did the quality of evidence.

Based on the summarized results, the constructs, and the population of interest, we identified several instruments that have the potential to be recommended for use in different healthcare disciplines. Future research measuring EBP attitudes, behavior, and self-efficacy should strive to build upon and further develop existing EBP instruments. In cases where new EBP instruments are being developed, the generation of questionnaire items should include qualitative methods involving members of the target population. In addition, future research should focus on reaching a clear articulation of and a shared conception of EBP constructs.

### Supplementary Information

**Additional file 1.** Search strategy.

**Additional file 2.** COSMIN criteria for good measurement properties.

**Additional file 3.** Characteristics of the included studies and participants.

**Additional file 4.** Results of quality assessment and measurement properties of the individual studies.

**Additional file 5.** The PRISMA 2020 checklist.

## Data Availability

Not applicable.

## Abbreviations

EBP: Evidence-based practice
COSMIN: Consensus-based Standards for the Selection of Health Measurement Instruments
PRISMA: Preferred Reporting Items for Systematic Reviews and Meta-Analyses
CREATE: Classification Rubric for Evidence-Based Practice Assessment Tools in Education
EFA: Exploratory factor analysis
CFA: Confirmatory factor analysis
CFI: Comparative fit index
RMSEA: Root mean square error of approximation
SRMR: Standardized square residual
ICC: Intraclass correlation coefficient
SEM: Standard error of measurement
LoA: Limits of agreement
SDC: Smallest detectable change
MIC: Minimal important change
