# Supplementary material for: Instruments measuring evidence-based practice behavior, attitudes, and self-efficacy among healthcare professionals: a systematic review of measurement properties
Source: Implement Sci. 2023 Sep 13;18:42. doi: 10.1186/s13012-023-01301-3 (PMC10500884; doi:10.1186/s13012-023-01301-3)
Supplement: Supplementary file 1 — Additional file 1. Search strategy. [file 13012_2023_1301_MOESM1_ESM.docx]

**Additional file 1:** Search strategy

# **Søkehistorikk**

**Database**: Ovid MEDLINE(R) ALL 1946 to October 01, 2020

**Dato**: 05.10.2020

**Antall treff**: 1341

| **#** | **Searches** | **Results** |
| --- | --- | --- |
| 1 | exp Evidence-Based Practice/ | 88831 |
| 2 | ((((evidence based or knowledge based or research based) adj2 (practice* or health care or healthcare or medicine or clinical practice or treatment or management)) or (EBP or EBM)) adj8 (instrument* or measur* or assess* or tool* or scale* or scoring* or score* or test* or survey* or questionnaire*)).tw,kw,kf. | 4149 |
| 3 | or/1-2 | 90929 |
| 4 | exp Health Personnel/ | 519637 |
| 5 | mental health services/ | 34134 |
| 6 | Social Workers/ | 649 |
| 7 | community mental health services/ | 18558 |
| 8 | "Attitude of Health Personnel"/ | 122563 |
| 9 | Caregivers/ | 37170 |
| 10 | Professional Practice/ | 16734 |
| 11 | professional competence/ | 24353 |
| 12 | clinical competence/ | 93983 |
| 13 | professional role/ | 14061 |
| 14 | nurse's role/ | 41033 |
| 15 | physician's role/ | 30119 |
| 16 | practice patterns, nurses'/ | 2747 |
| 17 | practice patterns, physicians'/ | 60521 |
| 18 | (((health or healthcare or health care) adj3 (provider* or personnel* or staff* or worker* or employee* or practitioner* or professional*)) or (medical* adj3 (staff* or personnel* or worker* or professional* or facult*)) or (hospital* adj3 (staff* or personnel* or worker*)) or (clinician* or care giver* or caregiver* or nurse* or nursing or hospitalist* or physician* or doctor* or occupational therapist* or physical therapist* or physiotherapist*)).tw,kw,kf. | 1406454 |
| 19 | or/4-18 | 1838732 |
| 20 | "surveys and questionnaires"/ | 469891 |
| 21 | self report/ | 33323 |
| 22 | "Weights and Measures"/ | 2571 |
| 23 | Psychological Tests/ | 36795 |
| 24 | Behavior Rating Scale/ | 407 |
| 25 | ((((evidence based or knowledge based or research based) adj2 (practice* or health care or healthcare or medicine or clinical practice or treatment or management)) or (EBP or EBM)) adj8 (instrument* or measur* or assess* or tool* or scale* or scoring* or score* or test* or survey* or questionnaire*)).tw,kw,kf. | 4149 |
| 26 | or/20-25 | 534351 |
| 27 | Psychometrics/ | 75919 |
| 28 | evaluation studies as topic/ | 121998 |
| 29 | "reproducibility of results"/ | 401488 |
| 30 | dimensional measurement accuracy/ | 566 |
| 31 | validation studies as topic/ | 2227 |
| 32 | data accuracy/ | 2594 |
| 33 | (psychometr* or propert* or valid* or reliab* or interpretab* or dimension* or consisten* or precision* or credib* or item* or domain*).tw,kw,kf. | 4200205 |
| 34 | or/27-33 | 4506569 |
| 35 | "Attitude of Health Personnel"/ | 122563 |
| 36 | attitude/ | 47538 |
| 37 | behavior/ | 29491 |
| 38 | Health Knowledge, Attitudes, Practice/ | 112496 |
| 39 | self concept/ | 56926 |
| 40 | self-assessment/ | 12563 |
| 41 | self efficacy/ | 20581 |
| 42 | Mental Competency/ | 8303 |
| 43 | professional competence/ | 24353 |
| 44 | clinical competence/ | 93983 |
| 45 | Work Performance/ | 870 |
| 46 | practice patterns, nurses'/ | 2747 |
| 47 | practice patterns, physicians'/ | 60521 |
| 48 | Professional Practice/ | 16734 |
| 49 | (belie* or behavio* or attitude* or self-efficacy or self-percepti* or self-confiden* or self-esteem or self-concept* or opinion* or competen* or apply* or applied or perform*).tw,kw,kf. | 5745628 |
| 50 | or/35-49 | 6029864 |
| 51 | 3 and 19 and 26 and 34 and 50 | 982 |
| 52 | (Implementation Self-efficacy for EBP or Implementation Self-efficacy for Evidence Based Practice or ISE4EBP or Evidence-based Practice Attitude Scale* or EBPAS-50 or EBPAS50 or EBPAS-36 or EBPAS36 or Assessing Competency in EBM or Assessing Competency in Evidence Based Medicine or ACE tool or ACE instrument or "Knowledge Attitudes Access and Confidence Evaluation" or "Knowledge, Attitudes, Access, and Confidence Evaluation" or KACE or Evidence-Based Practice Confidence or EBP Beliefs Scale or Evidence Based Practice Beliefs Scale or EBP Implementation Scale or Evidence Based Practice Implementation Scale or Evidence Based Implementation scale or evidence based practice questionnaire or EBPQ).tw,kw,kf. | 171 |
| 53 | ((((evidence based or knowledge based or research based) adj3 (practice* or health care or healthcare or medicine or clinical practice or treatment or management)) or (EBP or EBM)) and (instrument* or measur* or assess* or tool* or scale* or scoring* or score* or test* or survey* or questionnaire* or domain* or psychometr* or propert* or valid* or reliab* or interpretab* or dimension* or consisten* or precision* or credib* or item*)).ti. | 975 |
| 54 | 50 and 53 | 538 |
| 55 | 51 or 52 or 54 | 1395 |
| 56 | limit 55 to (danish or english or norwegian or swedish) | 1341 |

**Database**: Ovid Embase <1974 to 2020 October 02>

**Dato**: 05.10.2020

**Antall treff**: 2089

| **#** | **Searches** | **Results** |
| --- | --- | --- |
| 1 | evidence based medicine/ | 111843 |
| 2 | evidence based practice/ | 66226 |
| 3 | ((((evidence based or knowledge based or research based) adj2 (practice* or health care or healthcare or medicine or clinical practice or treatment or management)) or (EBP or EBM)) adj8 (instrument* or measur* or assess* or tool* or scale* or scoring* or score* or test* or survey* or questionnaire*)).tw,kw. | 5552 |
| 4 | or/1-3 | 179277 |
| 5 | exp health care personnel/ | 1576389 |
| 6 | social worker/ | 11729 |
| 7 | exp professional practice/ | 360747 |
| 8 | exp health personnel attitude/ | 185042 |
| 9 | social worker attitude/ | 88 |
| 10 | professional competence/ | 32167 |
| 11 | nursing competence/ | 1012 |
| 12 | caregiver/ | 83139 |
| 13 | (((health or healthcare or health care) adj3 (provider* or personnel* or staff* or worker* or employee* or practitioner* or professional*)) or (medical* adj3 (staff* or personnel* or worker* or professional* or facult*)) or (hospital* adj3 (staff* or personnel* or worker*)) or (clinician* or care giver* or caregiver* or nurse* or nursing or hospitalist* or physician* or doctor* or occupational therapist* or physical therapist* or physiotherapist*)).tw,kw. | 1788317 |
| 14 | or/5-13 | 2910241 |
| 15 | exp questionnaire/ | 725038 |
| 16 | behavior assessment/ | 8160 |
| 17 | attitude assessment/ | 1338 |
| 18 | individual behavior assessment/ | 298 |
| 19 | self-concept assessment/ | 370 |
| 20 | measurement/ | 96687 |
| 21 | performance measurement system/ | 4150 |
| 22 | attitude scale/ | 423 |
| 23 | rating scale/ | 112204 |
| 24 | scoring system/ | 262063 |
| 25 | ((((evidence based or knowledge based or research based) adj2 (practice* or health care or healthcare or medicine or clinical practice or treatment or management)) or (EBP or EBM)) adj8 (instrument* or measur* or assess* or tool* or scale* or scoring* or score* or test* or survey* or questionnaire*)).tw,kw. | 5552 |
| 26 | or/15-25 | 1139248 |
| 27 | psychometry/ | 62482 |
| 28 | exp reliability/ | 185048 |
| 29 | exp validity/ | 101707 |
| 30 | measurement accuracy/ | 40552 |
| 31 | dimensional measurement accuracy/ | 523 |
| 32 | measurement precision/ | 12536 |
| 33 | reproducibility/ | 220132 |
| 34 | measurement repeatability/ | 6215 |
| 35 | test retest reliability/ | 17668 |
| 36 | (psychometr* or propert* or valid* or reliab* or interpretab* or dimension* or consisten* or precision* or credib* or item* or domain*).tw,kw. | 5017413 |
| 37 | or/27-36 | 5193811 |
| 38 | behavior/ | 154049 |
| 39 | attitude/ | 63310 |
| 40 | behavior assessment/ | 8160 |
| 41 | attitude assessment/ | 1338 |
| 42 | individual behavior assessment/ | 298 |
| 43 | self-concept assessment/ | 370 |
| 44 | attitude scale/ | 423 |
| 45 | employee attitude/ | 814 |
| 46 | exp health personnel attitude/ | 185042 |
| 47 | exp professional practice/ | 360747 |
| 48 | social worker attitude/ | 88 |
| 49 | professional competence/ | 32167 |
| 50 | self concept/ | 90354 |
| 51 | competence/ | 25061 |
| 52 | clinical competence/ | 61369 |
| 53 | nursing competence/ | 1012 |
| 54 | performance/ | 33455 |
| 55 | job performance/ | 16801 |
| 56 | performance measurement system/ | 4150 |
| 57 | task performance/ | 143474 |
| 58 | self esteem/ | 22580 |
| 59 | implicit self esteem/ | 20 |
| 60 | explicit self esteem/ | 18 |
| 61 | (belie* or behavio* or attitude* or self-efficacy or self-percepti* or self-confiden* or self-esteem or self-concept* or opinion* or competen* or apply* or applied or perform*).tw,kw. | 7460189 |
| 62 | or/38-61 | 8037601 |
| 63 | 4 and 14 and 26 and 37 and 62 | 1675 |
| 64 | (Implementation Self-efficacy for EBP or Implementation Self-efficacy for Evidence Based Practice or ISE4EBP or Evidence-based Practice Attitude Scale* or EBPAS-50 or EBPAS50 or EBPAS-36 or EBPAS36 or Assessing Competency in EBM or Assessing Competency in Evidence Based Medicine or ACE tool or ACE instrument or "Knowledge Attitudes Access and Confidence Evaluation" or "Knowledge, Attitudes, Access, and Confidence Evaluation" or KACE or Evidence-Based Practice Confidence or EBP Beliefs Scale or Evidence Based Practice Beliefs Scale or EBP Implementation Scale or Evidence Based Practice Implementation Scale or Evidence Based Implementation scale or evidence based practice questionnaire or EBPQ).tw,kw. | 194 |
| 65 | ((((evidence based or knowledge based or research based) adj3 (practice* or health care or healthcare or medicine or clinical practice or treatment or management)) or (EBP or EBM)) and (instrument* or measur* or assess* or tool* or scale* or scoring* or score* or test* or survey* or questionnaire* or domain* or psychometr* or propert* or valid* or reliab* or interpretab* or dimension* or consisten* or precision* or credib* or item*)).ti. | 1137 |
| 66 | 62 and 65 | 627 |
| 67 | 63 or 64 or 66 | 2178 |
| 68 | limit 67 to (danish or english or norwegian or swedish) | 2089 |

**Database**: Ovid APA PsycInfo <1806 to September Week 4 2020>

**Dato**: 05.10.2020

**Antall treff**: 752

| **#** | **Searches** | **Results** |
| --- | --- | --- |
| 1 | evidence based practice/ | 18093 |
| 2 | ((((evidence based or knowledge based or research based) adj2 (practice* or health care or healthcare or medicine or clinical practice or treatment or management)) or (EBP or EBM)) adj8 (instrument* or measur* or assess* or tool* or scale* or scoring* or score* or test* or survey* or questionnaire*)).tw. | 2011 |
| 3 | 1 or 2 | 18883 |
| 4 | exp health personnel/ | 163403 |
| 5 | professional personnel/ | 5287 |
| 6 | exp therapists/ | 40450 |
| 7 | exp counselors/ | 18877 |
| 8 | exp psychologists/ | 32285 |
| 9 | exp social workers/ | 12496 |
| 10 | clinical practice/ | 20032 |
| 11 | exp professional role/ | 3425 |
| 12 | clinicians/ | 10377 |
| 13 | caregivers/ | 29042 |
| 14 | exp Health Personnel Attitudes/ | 23670 |
| 15 | counselor attitudes/ | 1901 |
| 16 | occupational attitudes/ | 2783 |
| 17 | psychologist attitudes/ | 1107 |
| 18 | professional competence/ | 7219 |
| 19 | (((health or healthcare or health care) adj3 (provider* or personnel* or staff* or worker* or employee* or practitioner* or professional*)) or (medical* adj3 (staff* or personnel* or worker* or professional* or facult*)) or (hospital* adj3 (staff* or personnel* or worker*)) or (clinician* or care giver* or caregiver* or nurse* or nursing or hospitalist* or physician* or doctor* or occupational therapist* or physical therapist* or physiotherapist*)).tw. | 398553 |
| 20 | or/4-19 | 533335 |
| 21 | exp testing/ | 217580 |
| 22 | measurement/ | 52818 |
| 23 | professional examinations/ | 1027 |
| 24 | professional measures/ | 42 |
| 25 | ((((evidence based or knowledge based or research based) adj2 (practice* or health care or healthcare or medicine or clinical practice or treatment or management)) or (EBP or EBM)) adj8 (instrument* or measur* or assess* or tool* or scale* or scoring* or score* or test* or survey* or questionnaire*)).tw. | 2011 |
| 26 | or/21-25 | 259453 |
| 27 | exp psychometrics/ | 192682 |
| 28 | (psychometr* or propert* or valid* or reliab* or interpretab* or dimension* or consisten* or precision* or credib* or item* or domain*).tw. | 986659 |
| 29 | or/27-28 | 1029846 |
| 30 | attitudes/ | 27385 |
| 31 | adult attitudes/ | 11711 |
| 32 | employee attitudes/ | 17797 |
| 33 | exp health personnel attitudes/ | 23670 |
| 34 | psychologist attitudes/ | 1107 |
| 35 | occupational attitudes/ | 2783 |
| 36 | counselor attitudes/ | 1901 |
| 37 | "work (attitudes toward)"/ | 6376 |
| 38 | self-efficacy/ | 23190 |
| 39 | self-perception/ | 21333 |
| 40 | self-concept/ | 43610 |
| 41 | self-confidence/ | 3695 |
| 42 | self-esteem/ | 25583 |
| 43 | self-regard/ | 168 |
| 44 | false beliefs/ | 1007 |
| 45 | behavior/ | 26252 |
| 46 | choice behavior/ | 18909 |
| 47 | exp planned behavior/ | 4550 |
| 48 | implicit attitudes/ | 1012 |
| 49 | explicit attitudes/ | 350 |
| 50 | performance/ | 23238 |
| 51 | exp job performance/ | 21069 |
| 52 | exp competence/ | 26035 |
| 53 | professional competence/ | 7219 |
| 54 | (belie* or behavio* or attitude* or self-efficacy or self-percepti* or self-confiden* or self-esteem or self-concept* or opinion* or competen* or apply* or applied or perform*).tw. | 2014274 |
| 55 | or/30-54 | 2081473 |
| 56 | 3 and 20 and 26 and 29 and 55 | 346 |
| 57 | (Implementation Self-efficacy for EBP or Implementation Self-efficacy for Evidence Based Practice or ISE4EBP or Evidence-based Practice Attitude Scale* or EBPAS-50 or EBPAS50 or EBPAS-36 or EBPAS36 or Assessing Competency in EBM or Assessing Competency in Evidence Based Medicine or ACE tool or ACE instrument or "Knowledge Attitudes Access and Confidence Evaluation" or "Knowledge, Attitudes, Access, and Confidence Evaluation" or KACE or Evidence-Based Practice Confidence or EBP Beliefs Scale or Evidence Based Practice Beliefs Scale or EBP Implementation Scale or Evidence Based Practice Implementation Scale or Evidence Based Implementation scale or evidence based practice questionnaire or EBPQ).tw. | 97 |
| 58 | ((((evidence based or knowledge based or research based) adj3 (practice* or health care or healthcare or medicine or clinical practice or treatment or management)) or (EBP or EBM)) and (instrument* or measur* or assess* or tool* or scale* or scoring* or score* or test* or survey* or questionnaire* or domain* or psychometr* or propert* or valid* or reliab* or interpretab* or dimension* or consisten* or precision* or credib* or item*)).ti. or (evidence* based* or ebp* or ebm* or ebn*).tm. | 785 |
| 59 | 55 and 58 | 532 |
| 60 | 56 or 57 or 59 | 765 |
| 61 | limit 60 to (danish or english or norwegian or swedish) | 752 |

**Database**: The Cochrane Library via Wiley

**Dato**: 05.10.2020

**Antall treff**: 144 (Cochrane Reviews: 1; Trials: 143)

| ID | Search | Hits |
| --- | --- | --- |
| #1 | MeSH descriptor: [Evidence-Based Practice] explode all trees | 1224 |
| #2 | ((((evidence NEXT based or knowledge NEXT based or research NEXT based) NEAR/2 (practice* or health NEXT care or healthcare or medicine or clinical practice or treatment or management)) or (EBP or EBM)) NEAR/8 (instrument* or measur* or assess* or tool* or scale* or scoring* or score* or test* or survey* or questionnaire*)):ti,ab,kw | 1033 |
| #3 | #1 or #2 | 2165 |
| #4 | MeSH descriptor: [Health Personnel] explode all trees | 8776 |
| #5 | MeSH descriptor: [Mental Health Services] this term only | 642 |
| #6 | MeSH descriptor: [Social Workers] this term only | 25 |
| #7 | MeSH descriptor: [Community Mental Health Services] this term only | 722 |
| #8 | MeSH descriptor: [Attitude of Health Personnel] this term only | 1916 |
| #9 | MeSH descriptor: [Professional Practice] this term only | 110 |
| #10 | MeSH descriptor: [Caregivers] this term only | 2094 |
| #11 | MeSH descriptor: [Professional Competence] this term only | 250 |
| #12 | MeSH descriptor: [Clinical Competence] this term only | 3254 |
| #13 | MeSH descriptor: [Professional Role] this term only | 209 |
| #14 | MeSH descriptor: [Nurse's Role] this term only | 318 |
| #15 | MeSH descriptor: [Physician's Role] this term only | 190 |
| #16 | MeSH descriptor: [Practice Patterns, Nurses'] this term only | 150 |
| #17 | MeSH descriptor: [Practice Patterns, Physicians'] this term only | 1214 |
| #18 | (((health or healthcare or health NEXT care) NEAR/3 (provider* or personnel* or staff* or worker* or employee* or practitioner* or professional*)) or (medical* NEAR/3 (staff* or personnel* or worker* or professional* or facult*)) or (hospital* NEAR/3 (staff* or personnel* or worker*)) or (clinician* or care NEXT giver* or caregiver* or nurse* or nursing or hospitalist* or physician* or doctor* or occupational NEXT therapist* or physical NEXT therapist* or physiotherapist*)):ti,ab,kw | 136325 |
| #19 | {OR #4-#18} | 139771 |
| #20 | MeSH descriptor: [Surveys and Questionnaires] this term only | 25160 |
| #21 | MeSH descriptor: [Self Report] this term only | 2236 |
| #22 | MeSH descriptor: [Weights and Measures] this term only | 36 |
| #23 | MeSH descriptor: [Psychological Tests] this term only | 1774 |
| #24 | MeSH descriptor: [Behavior Rating Scale] this term only | 11 |
| #25 | ((((evidence NEXT based or knowledge NEXT based or research NEXT based) NEAR/2 (practice* or health NEXT care or healthcare or medicine or clinical practice or treatment or management)) or (EBP or EBM)) NEAR/8 (instrument* or measur* or assess* or tool* or scale* or scoring* or score* or test* or survey* or questionnaire*)):ti,ab,kw | 1033 |
| #26 | {OR #20-#25} | 29670 |
| #27 | MeSH descriptor: [Psychometrics] this term only | 2811 |
| #28 | MeSH descriptor: [Evaluation Studies as Topic] this term only | 3702 |
| #29 | MeSH descriptor: [Reproducibility of Results] this term only | 10696 |
| #30 | MeSH descriptor: [Dimensional Measurement Accuracy] this term only | 14 |
| #31 | MeSH descriptor: [Validation Studies as Topic] this term only | 21 |
| #32 | MeSH descriptor: [Data Accuracy] this term only | 27 |
| #33 | (psychometr* or propert* or valid* or reliab* or interpretab* or dimension* or consisten* or precision* or credib* or item* or domain*):ti,ab,kw | 189533 |
| #34 | {OR #27-#33} | 197998 |
| #35 | MeSH descriptor: [Attitude of Health Personnel] this term only | 1916 |
| #36 | MeSH descriptor: [Attitude] this term only | 1053 |
| #37 | MeSH descriptor: [Behavior] this term only | 854 |
| #38 | MeSH descriptor: [Health Knowledge, Attitudes, Practice] this term only | 5897 |
| #39 | MeSH descriptor: [Self Concept] this term only | 2351 |
| #40 | MeSH descriptor: [Self-Assessment] this term only | 731 |
| #41 | MeSH descriptor: [Self Efficacy] this term only | 3111 |
| #42 | MeSH descriptor: [Mental Competency] this term only | 82 |
| #43 | MeSH descriptor: [Professional Competence] this term only | 250 |
| #44 | MeSH descriptor: [Clinical Competence] this term only | 3254 |
| #45 | MeSH descriptor: [Work Performance] this term only | 42 |
| #46 | MeSH descriptor: [Practice Patterns, Nurses'] this term only | 150 |
| #47 | MeSH descriptor: [Practice Patterns, Physicians'] this term only | 1214 |
| #48 | MeSH descriptor: [Professional Practice] this term only | 110 |
| #49 | (belie* or behavio* or attitude* or self NEXT efficacy or self NEXT percepti* or self NEXT confiden* or self NEXT esteem or self NEXT concept* or opinion* or competen* or apply* or applied or perform*):ti,ab,kw | 467833 |
| #50 | {OR #35-#49} | 468747 |
| #51 | #3 AND #19 AND #26 AND #34 AND #50 | 97 |
| #52 | ((Implementation NEXT Self NEXT efficacy NEXT for NEXT EBP) or (Implementation NEXT Self NEXT efficacy NEXT for NEXT Evidence NEXT Based NEXT Practice) or ISE4EBP or (Evidence NEXT based NEXT Practice NEXT Attitude NEXT Scale*) or (EBPAS NEXT 50) or EBPAS50 or (EBPAS NEXT 36) or EBPAS36 or (Assessing NEXT Competency NEXT in NEXT EBM) or (Assessing NEXT Competency NEXT in NEXT Evidence NEXT Based NEXT Medicine) or (ACE NEXT tool) or (ACE NEXT instrument) or (Knowledge NEXT Attitudes NEXT Access NEXT "and" NEXT Confidence NEXT Evaluation) or (Knowledge NEXT Attitudes NEXT Access NEXT "and" NEXT Confidence NEXT Evaluation) or KACE or (Evidence NEXT Based NEXT Practice NEXT Confidence) or (EBP NEXT Beliefs NEXT Scale) or (Evidence NEXT Based NEXT Practice NEXT Beliefs NEXT Scale) or (EBP NEXT Implementation NEXT Scale) or (Evidence NEXT Based NEXT Practice NEXT Implementation NEXT Scale) or (Evidence NEXT Based NEXT Implementation NEXT scale) or (evidence NEXT based NEXT practice NEXT questionnaire) or EBPQ):ti,ab,kw | 24 |
| #53 | ((((evidence NEXT based or knowledge NEXT based or research NEXT based) NEAR/3 (practice* or health NEXT care or healthcare or medicine or clinical practice or treatment or management)) or (EBP or EBM)) and (instrument* or measur* or assess* or tool* or scale* or scoring* or score* or test* or survey* or questionnaire* or domain* or psychometr* or propert* or valid* or reliab* or interpretab* or dimension* or consisten* or precision* or credib* or item*)):ti | 69 |
| #54 | #53 AND #50 | 35 |
| #55 | #51 or #52 or #54 | 144 |

**Database**: Cinahl via EBSCOhost

**Dato**: 05.10.2020

**Antall treff**: 1417

| **ID#** | **Search Terms** | **Results** |
| --- | --- | --- |
| S1 | (MH "Professional Practice, Evidence-Based+") | 78,368 |
| S2 | (MH "Professional Practice, Research-Based+") | 4,998 |
| S3 | TI ( (((("evidence based" or "knowledge based" or "research based") N2 (practice* or "health care" or healthcare or medicine or "clinical practice" or treatment or management)) or (EBP or EBM)) N8 (instrument* or measur* or assess* or tool* or scale* or scoring* or score* or test* or survey* or questionnaire*)) ) OR AB ( (((("evidence based" or "knowledge based" or "research based") N2 (practice* or "health care" or healthcare or medicine or "clinical practice" or treatment or management)) or (EBP or EBM)) N8 (instrument* or measur* or assess* or tool* or scale* or scoring* or score* or test* or survey* or questionnaire*)) ) | 3,221 |
| S4 | S1 OR S2 OR S3 | 79,719 |
| S5 | (MH "Health Personnel+") | 574,312 |
| S6 | (MH "Attitude of Health Personnel+") | 100,117 |
| S7 | (MH "Professional Role+") | 110,558 |
| S8 | (MH "Professional Practice") | 16,895 |
| S9 | (MH "Professional Competence") | 17,149 |
| S10 | (MH "Clinical Competence") | 42,251 |
| S11 | TI ( (((health or healthcare or "health care") N3 (provider* or personnel* or staff* or worker* or employee* or practitioner* or professional*)) or (medical* N3 (staff* or personnel* or worker* or Professional* or facult*)) or (hospital* N3 (staff* or personnel* or worker*)) or (clinician* or "care giver*" or caregiver* or nurse* or nursing or hospitalist* or physician* or doctor* or "occupational therapist*" or physical therapist* or physiotherapist*)) ) OR AB ( (((health or healthcare or "health care") N3 (provider* or personnel* or staff* or worker* or employee* or practitioner* or professional*)) or (medical* N3 (staff* or personnel* or worker* or Professional* or facult*)) or (hospital* N3 (staff* or personnel* or worker*)) or (clinician* or "care giver*" or caregiver* or nurse* or nursing or hospitalist* or physician* or doctor* or "occupational therapist*" or "physical therapist*" or physiotherapist*)) ) | 970,189 |
| S12 | S5 OR S6 OR S7 OR S8 OR S9 OR S10 OR S11 | 1,381,039 |
| S13 | (MH "Questionnaires+") | 414,135 |
| S14 | (MH "Attitude Measures") | 9,548 |
| S15 | (MH "Behavior Rating Scales") | 2,723 |
| S16 | (MH "Scales") | 346,568 |
| S17 | (MH "Psychological Tests") | 82,911 |
| S18 | TI ( (((("evidence based" or "knowledge based" or "research based") N2 (practice* or "health care" or healthcare or medicine or "clinical practice" or treatment or management)) or (EBP or EBM)) N8 (instrument* or measur* or assess* or tool* or scale* or scoring* or score* or test* or survey* or questionnaire*)) ) OR AB ( (((("evidence based" or "knowledge based" or "research based") N2 (practice* or "health care" or healthcare or medicine or "clinical practice" or treatment or management)) or (EBP or EBM)) N8 (instrument* or measur* or assess* or tool* or scale* or scoring* or score* or test* or survey* or questionnaire*)) ) | 3,221 |
| S19 | S13 OR S14 OR S15 OR S16 OR S17 OR S18 | 713,600 |
| S20 | (MH "Psychometrics") | 26,706 |
| S21 | (MH "Measurement Issues and Assessments") | 2,839 |
| S22 | (MH "Measurement Error") | 797 |
| S23 | (MH "Precision") | 646 |
| S24 | (MH "Reliability and Validity+") | 252,993 |
| S25 | TI ( (psychometr* or propert* or valid* or reliab* or interpretab* or dimension* or consisten* or precision* or credib* or item* or domain*) ) OR AB ( (psychometr* or propert* or valid* or reliab* or interpretab* or dimension* or consisten* or precision* or credib* or item* or domain*) ) | 582,664 |
| S26 | S20 OR S21 OR S22 OR S23 OR S24 OR S25 | 756,627 |
| S27 | (MH "Attitude of Health Personnel+") | 100,117 |
| S28 | (MH "Attitude") | 16,057 |
| S29 | (MH "Behavior") | 19,469 |
| S30 | (MH "Employee Attitudes") | 4,345 |
| S31 | (MH "Self-Efficacy") | 21,454 |
| S32 | (MH "Self Assessment") | 9,562 |
| S33 | (MH "Behavior Rating Scales") | 2,723 |
| S34 | (MH "Attitude Measures") | 9,548 |
| S35 | (MH "Self Concept") | 31,979 |
| S36 | (MH "Professional Competence") or (MH "Professional Practice") | 33,433 |
| S37 | (MH "Clinical Competence") | 42,251 |
| S38 | TI ( (belie* or behavio* or attitude* or "self-efficacy" or "self-percepti*" or "self-confiden*" or "self-esteem" or "self-concept*" or opinion* or competen* or apply* or applied or perform*) ) OR AB ( (belie* or behavio* or attitude* or "self-efficacy" or "self-percepti*" or "self-confiden*" or "self-esteem" or "self-concept*" or opinion* or competen* or apply* or applied or perform*) ) | 1,152,245 |
| S39 | S27 OR S28 OR S29 OR S30 OR S31 OR S32 OR S33 OR S34 OR S35 OR S36 OR S37 OR S38 | 1,303,772 |
| S40 | S4 AND S12 AND S19 AND S26 AND S39 | 1,151 |
| S41 | TI ( (“Implementation Self-efficacy for EBP” or “Implementation Self-efficacy for Evidence Based Practice” or ISE4EBP or “Evidence-based Practice Attitude Scale*” or “EBPAS-50” or EBPAS50 or “EBPAS-36” or EBPAS36 or “Assessing Competency in EBM” or “Assessing Competency in Evidence Based Medicine” or “ACE tool” or “ACE instrument” or "Knowledge Attitudes Access and Confidence Evaluation" or "Knowledge, Attitudes, Access, and Confidence Evaluation" or KACE or “Evidence-Based Practice Confidence” or “EBP Beliefs Scale” or “Evidence Based Practice Beliefs Scale” or “EBP Implementation Scale” or “Evidence Based Practice Implementation Scale” or “Evidence Based Implementation scale” or “evidence based practice questionnaire” or EBPQ) ) OR AB ( (“Implementation Self-efficacy for EBP” or “Implementation Self-efficacy for Evidence Based Practice” or ISE4EBP or “Evidence-based Practice Attitude Scale*” or “EBPAS-50” or EBPAS50 or “EBPAS-36” or EBPAS36 or “Assessing Competency in EBM” or “Assessing Competency in Evidence Based Medicine” or “ACE tool” or “ACE instrument” or "Knowledge Attitudes Access and Confidence Evaluation" or "Knowledge, Attitudes, Access, and Confidence Evaluation" or KACE or “Evidence-Based Practice Confidence” or “EBP Beliefs Scale” or “Evidence Based Practice Beliefs Scale” or “EBP Implementation Scale” or “Evidence Based Practice Implementation Scale” or “Evidence Based Implementation scale” or “evidence based practice questionnaire” or EBPQ) ) | 155 |
| S42 | TI (((("evidence based" or "knowledge based" or "research based") N3 (practice* or health care or healthcare or medicine or clinical practice or treatment or management)) or (EBP or EBM)) and (instrument* or measur* or assess* or tool* or scale* or scoring* or score* or test* or survey* or questionnaire* or domain* or psychometr* or propert* or valid* or reliab* or interpretab* or dimension* or consisten* or precision* or credib* or item*)) | 934 |
| S43 | S39 AND S42 | 411 |
| S44 | S40 OR S41 OR S43 | 1,455 |
| S45 | S40 OR S41 OR S43  Limiters - Language: Danish, English, Norwegian, Swedish | 1,417 |

**Database**: AMED

**Dato**: 05.10.2020

**Antall treff**: 69

1 Evidence based medicine/ (2956)

2 ((((evidence based or knowledge based or research based) adj2 (practice* or health care or healthcare or medicine or clinical practice or treatment or management)) or (EBP or EBM)) adj8 (instrument* or measur* or assess* or tool* or scale* or scoring* or score* or test* or survey* or questionnaire*)).tw. (285)

3 1 or 2 (3068)

4 exp health personnel/ (6639)

5 Caregivers/ (3168)

6 "attitude of health personnel"/ (3598)

7 professional practice/ (7764)

8 Professional competence/ (1577)

9 clinical competence/ (393)

10 Social work/ (360)

11 Mental health services/ (862)

12 Community mental health services/ (845)

13 nurses role/ (517)

14 Physicians role/ (455)

15 (((health or healthcare or health care) adj3 (provider* or personnel* or staff* or worker* or employee* or practitioner* or professional*)) or (medical* adj3 (staff* or personnel* or worker* or professional* or facult*)) or (hospital* adj3 (staff* or personnel* or worker*)) or (clinician* or care giver* or caregiver* or nurse* or nursing or hospitalist* or physician* or doctor* or occupational therapist* or physical therapist* or physiotherapist*)).tw. (45992)

16 4 or 5 or 6 or 7 or 8 or 9 or 10 or 11 or 12 or 13 or 14 or 15 (53789)

17 Questionnaires/ (5131)

18 Measurement/ (2196)

19 personality assessment/ (37)

20 Psychological Tests/ (970)

21 ((((evidence based or knowledge based or research based) adj2 (practice* or health care or healthcare or medicine or clinical practice or treatment or management)) or (EBP or EBM)) adj8 (instrument* or measur* or assess* or tool* or scale* or scoring* or score* or test* or survey* or questionnaire*)).tw. (285)

22 17 or 18 or 19 or 20 or 21 (8438)

23 Psychometrics/ (1280)

24 Standards/ (16667)

25 "consistency and reliability"/ (1899)

26 "reproducibility of results"/ (2564)

27 (psychometr* or propert* or valid* or reliab* or interpretab* or dimension* or consisten* or precision* or credib* or item* or domain*).tw. (40355)

28 23 or 24 or 25 or 26 or 27 (53124)

29 "attitude of health personnel"/ (3598)

30 Attitude/ (2057)

31 behavior/ (1378)

32 "behavior and behavior mechanisms"/ (8)

33 adaptation psychological/ (4228)

34 Orientation/ (70)

35 Self concept/ (1932)

36 Perceived exertion/ (218)

37 Self assessment/ (1077)

38 Self efficacy/ (736)

39 Professional competence/ or Professional practice/ (9182)

40 clinical competence/ (393)

41 (belie* or behavio* or attitude* or self-efficacy or self-percepti* or self-confiden* or self-esteem or self-concept* or opinion* or competen* or apply* or applied or perform*).tw. (79929)

42 29 or 30 or 31 or 32 or 33 or 34 or 35 or 36 or 37 or 38 or 39 or 40 or 41 (89549)

43 3 and 16 and 22 and 28 and 42 (48)

44 (Implementation Self-efficacy for EBP or Implementation Self-efficacy for Evidence Based Practice or ISE4EBP or Evidence-based Practice Attitude Scale* or EBPAS-50 or EBPAS50 or EBPAS-36 or EBPAS36 or Assessing Competency in EBM or Assessing Competency in Evidence Based Medicine or ACE tool or ACE instrument or "Knowledge Attitudes Access and Confidence Evaluation" or "Knowledge, Attitudes, Access, and Confidence Evaluation" or KACE or Evidence-Based Practice Confidence or EBP Beliefs Scale or Evidence Based Practice Beliefs Scale or EBP Implementation Scale or Evidence Based Practice Implementation Scale or Evidence Based Implementation scale or evidence based practice questionnaire or EBPQ).tw. (6)

45 ((((evidence based or knowledge based or research based) adj3 (practice* or health care or healthcare or medicine or clinical practice or treatment or management)) or (EBP or EBM)) and (instrument* or measur* or assess* or tool* or scale* or scoring* or score* or test* or survey* or questionnaire* or domain* or psychometr* or propert* or valid* or reliab* or interpretab* or dimension* or consisten* or precision* or credib* or item*)).ti. (45)

46 42 and 45 (33)

47 43 or 44 or 46 (71)

48 limit 47 to (danish or english or norwegian or swedish) (69)

**Database**: Google Scholar via Publish or Perish

**Dato**: 05.10.2020

**Antall treff**: 366

"evidence based practice" personnel|professionals|nurses|physicians|doctors|"occupational therapists"|"physical therapists"|physiotherapists Instrument|scale|test|psychometric|properties|validity|reliability|items|domains attitude|behavior|"self efficacy"

OR

intitle:"evidence based practice" intitle:instrument|measuring|measurement|tool|scale|survey|psychometric|validity|reliability|items|domains|dimensions|assessment|assessing|properties|consistency|questionnaire intitle:attitude|behavior|"self efficacy"

**Database**: HaPI

**Dato**: 05.10.2020

**Antall treff**: 311

1 (evidence based or knowledge based or research based or ebp or ebm).mp. (362)

2 (psychometr* or propert* or valid* or reliab* or interpretab* or dimension* or consisten* or precision* or credib*or item* or domain* or instrument* or measur* or assess* or tool* or scale* or scoring* or score* or test* or survey* or questionnaire*).mp. (172621)

3 1 and 2 (311)

**Database**: Web of Science

**Dato**: 05.10.2020

**Antall treff**: 1042

**S1:** (505): TS=((((("evidence  based" or "knowledge  based" or "research  based") NEAR/2 (practice* or "health care" or healthcare or medicine or "clinical practice" or treatment or management)) or EBP or EBM) NEAR/8  (instrument* or measure* or assess* or tool* or scale* or scoring* or score* or test* or survey* or questionnaire*))) AND TS=(((psychometr* or propert* or valid* or reliab* or interpretab* or dimension* or consisten*  or precision* or  credib* or item* or domain*))) AND TS=(((((health or healthcare  or "health care") NEAR/3 (provider* or personnel* or staff* or worker* or employee* or practitioner* or professional*)) or (medical* NEAR/3 (staff* or personnel* or worker* or Professional* or facult*)) or (hospital* NEAR/3 (staff* or personnel* or worker*))  or (clinician* or care giver* or caregiver* or nurse* or nursing or hospitalist* or physician* or doctor* or "occupational therapist*" or "physical therapist*" or physiotherapist*)))) AND TS=(((belie* or behavio* or attitude* or "self-efficacy" or "self-percepti*" or "self-confiden*" or "self-esteem" or "self-concept*" or opinion* or competen* or apply* or applied or perform*)))

**S2:** (169): **TOPIC:**  ((("Implementation Self-efficacy for EBP" or "Implementation Self-efficacy for Evidence Based Practice" or ISE4EBP or "Evidence-based Practice Attitude Scale*" or "EBPAS-50" or EBPAS50 or "EBPAS-36" or EBPAS36 or "Assessing Competency in EBM" or "Assessing Competency in Evidence Based Medicine" or "ACE tool" or "ACE instrument" or "Knowledge Attitudes Access and Confidence Evaluation" or "Knowledge, Attitudes, Access, and Confidence Evaluation" or KACE or "Evidence-Based Practice Confidence" or "EBP Beliefs Scale" or "Evidence Based Practice Beliefs Scale" or "EBP Implementation Scale" or "Evidence Based Practice Implementation Scale" or "Evidence Based Implementation scale" or "evidence based practice questionnaire" or EBPQ)))

**S3:** (635): TI=((((("evidence  based"  or  "knowledge  based"  or  "research  based")  AND  (practice* or "health care" or healthcare or medicine or clinical practice or treatment or management))  or  (EBP or EBM))  AND  (instrument* or measur* or assess* or tool* or scale* or scoring* or score* or test* or survey* or questionnaire* or domain* or psychometr* or propert* or valid* or reliab* or interpretab* or dimension* or consisten* or precision* or credib* or item*)))  AND  TS=(((belie*  or  behavio*  or  attitude*  or  "self-efficacy"  or  "self-percepti*"  or  "self-confiden*"  or  "self-esteem"  or  "self-concept*" or opinion* or  competen* or  apply* or applied or perform*)))

**S4:** S1 or S2 or S3: 1064

**S5**: Limit: language: English, Norwegian, Swedish, Danish: 1042

**Updated search December 2022:**

The number of studies retrieved in the search with a time limit of 2020 – 2022/current: MEDLINE (333), EMBASE (547), PsycINFO (165), AMED (10), Web of Science (175), CINAHL (311), Cochrane Library (33), Google Scholar (300).
