# Supplementary material for: Instruments measuring evidence-based practice behavior, attitudes, and self-efficacy among healthcare professionals: a systematic review of measurement properties
Source: Implement Sci. 2023 Sep 13;18:42. doi: 10.1186/s13012-023-01301-3 (PMC10500884; doi:10.1186/s13012-023-01301-3)
Supplement: Supplementary file 2 — Additional file 2. COSMIN criteria for good measurement properties. [file 13012_2023_1301_MOESM2_ESM.docx]

**Additional file 2**: COSMINs criteria for good measurement properties

**Criteria structural validity, Internal consistency, Reliability, Measurement error
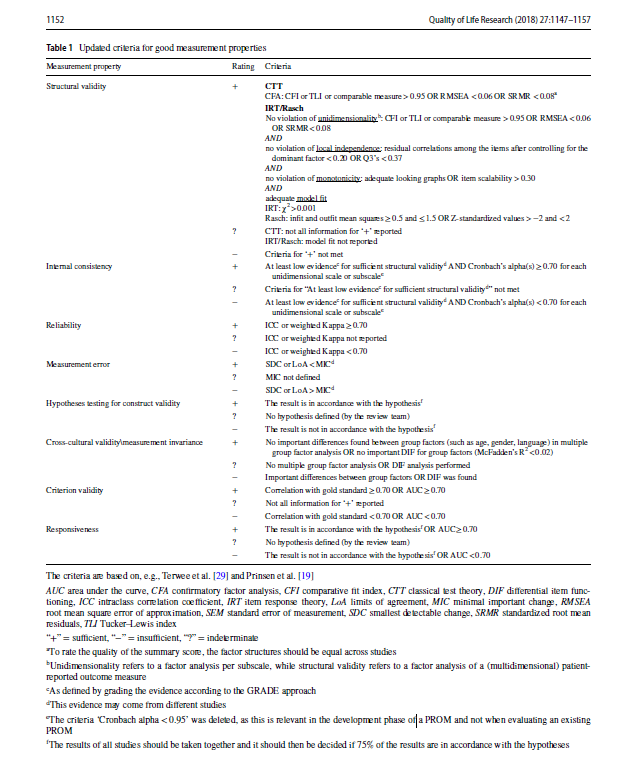
 (1)**:

**Criteria EFA/ PCA (2)**


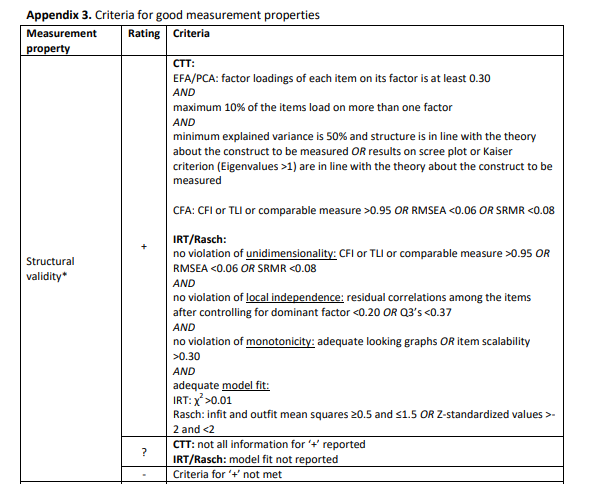


**Criteria PSI (3):**

Values ≥0.70 and ≥0.90 were considered to indicate adequate reliability for group-level and individual-level applications, respectively. (3)

**Additional file 3**: Characteristics of the included studies and participants.

**Additional file 4**: Results of quality assessment and measurement properties of the individual studies

1. Prinsen CAC, Mokkink LB, Bouter LM, Alonso J, Patrick DL, de Vet HCW, et al. COSMIN guideline for systematic reviews of patient-reported outcome measures. Qual Life Res. 2018;27(5):1147-57.

2. Elsman EBM, Mokkink LB, Langendoen-Gort M, Rutters F, Beulens J, Elders PJM, et al. Systematic review on the measurement properties of diabetes-specific patient-reported outcome measures (PROMs) for measuring physical functioning in people with type 2 diabetes. BMJ Open Diabetes Res Care. 2022;10(3).

3. Oude Voshaar MA, Ten Klooster PM, Glas CA, Vonkeman HE, Taal E, Krishnan E, et al. Validity and measurement precision of the PROMIS physical function item bank and a content validity-driven 20-item short form in rheumatoid arthritis compared with traditional measures. Rheumatology (Oxford). 2015;54(12):2221-9.
