# Supplementary material for: Instruments measuring evidence-based practice behavior, attitudes, and self-efficacy among healthcare professionals: a systematic review of measurement properties
Source: Implement Sci. 2023 Sep 13;18:42. doi: 10.1186/s13012-023-01301-3 (PMC10500884; doi:10.1186/s13012-023-01301-3)
Supplement: Supplementary file 3 — Additional file 3. Characteristics of the included studies and participants. [file 13012_2023_1301_MOESM3_ESM.docx]

| **Additional file 3** | | | | | | | | | |
| --- | --- | --- | --- | --- | --- | --- | --- | --- | --- |
| Characteristics of the included studies and participants. | | | | | | | | | |
|  |  |  |  |  |  |  |  |  |  |
| Instrument (abbreviation) | Reference | Construct(s) | Status | Country-  Language | EBP steps | Items/  Scale type | Dimensionality/  Subscales | Population | Sample size |
|  |  |  |  |  |  |  |  |  |  |
| Evidence based practice attitude scale (EBPAS) | Aarons, 2004 (1) | EBP attitudes | Development | USA- English | Ask, search, appraise, integrate | 15 items/ 5-point scale | Multidimensional/ Requirements, appeal, openness, divergence | Mental health workers | 322 |
| “ | Aarons et al., 2007 (2) | “ | Validation | “ | “ | “ | “ | Mental health workers | 221 |
| “ | Aarons et al., 2010 (3) | “ | Validation | “ | “ | “ | “ | Mental health workers | 1089 |
| “ | Maessen et al., 2019 (4) | “ | Translation, validation | The Netherlands-Dutch | “ | “ | “ | Nurses | 273 |
| “ | Melas et al., 2012 (5) | “ | Translation, validation | Greece-Greek | “ | “ | “ | Medical doctors | 534 |
| “ | Skavberg Roaldsen & Halvarsson, 2019 (6) | “ | Translation, validation | Sweden-Swedish | “ | “ | “ | Physiotherapists | 55 |
| “ | Egeland et al., 2016 (7) | “ | Translation, validation | Norway- Norwegian | “ | “ | “ | Psychologists, nurses, medical doctors, social workers, Physiotherapists | 294 |
| “ | Santesson et al., 2019 (8) | “ | Validation | Sweden-Swedish | “ | “ | “ | Mental health workers | 565 |
| “ | Ashcraft et al., 2011 (9) | “ | Validation | USA-English | “ | “ | “ | Mental health workers, others | 543 |
| “ | Baumann et al., 2022 (10) | “ | Translation, validation | Brazil- Portugese | “ | 14 items/ 5-point scale | “ | Psychology, medicine, other | 362 |
| “ | Ayhan Baser et al., 2021 (11) | “ | Translation, validation | Turkey- Turkish | “ | “ | “ | Family medicine residents | 151 |
| “ | Van Giang et al., 2021 (12) | “ | Translation, validation | Vietnam- Vietnamese | “ | “ | “ | Nurses, miwifes | 353 |
|  |  |  |  |  |  |  |  |  |  |
| The Evidence Based Practice Attitude Scale-50 (EBPAS-50) | Aarons et al., 2012 (13) | EBP attitudes | Development, validation | USA-English | Ask, search, appraise, integrate | 50 items/ 5-point scale | Multidimensional/ 12 subscales | Mental health workers | 420 |
| “ | Yildiz et al., 2018 (14) | “ | Translation, validation | Turkey-Turkish | “ | “ | “ | Nurses | 250 |
|  |  |  |  |  |  |  |  |  |  |
| The Evidence-based Practice Attitude Scale36 (EBPAS-36) | Rye et al., 2017 (15) | EBP attitudes | Adaption, Translation, validation | Norway- Norwegian | Ask, search, appraise, integrate | 36 items/ 5-point scale | Multidimensional/ 12 subscales | Psychologists, nurses, others | 838 |
| “ | Szota et al., 2021 (16) | “ | Translation, validation | Germany- German | “ | “ | “ | Psychotherapists | 599 |
|  |  |  |  |  |  |  |  |  |  |
| The evidence-based practice questionnaire (EBPQ) | Upton & Upton, 2006 (17) | EBP Attitudes, behavior, knowledge/skills | Development, validation | Wales- English | Ask, search, appraise, integrate, evaluate | 24 items/ 7-point scale | Multidimensional/ EBP attitudes, EBP practice, EBP knowledge/ skills | Nurses | 751 |
| “ | Son et al., 2014 (18) | “ | Translation, validation | Korea- Korean | “ | “ | “ | Nurses | 801 |
| “ | Tomotaki et al., 2018 (19) | “ | Translation, validation | Japan- Japanese | “ | 18 items/ 7-point scale | Multidimensional/ EBP attitudes, EBP practice, EBP knowledge/ skills 1, EBP knowledge/ skills 2 | Nurses | 501 |
| “ | Yang et al., 2019 (20) | “ | Translation, validation | China- Chinese | “ | 22 items/ 7-point scale | “ | Nurses | 810 |
|  |  |  |  |  |  |  |  |  |  |
| “ | Zaybak et al., 2017 (21) | “ | Translation, validation | Turkey- Turkish | “ | “ | “ | Nurses | 123 |
| “ | Sese-Abad et al., 2014 (22) | “ | Validation | Spain- Spanish | “ | 19 items/ 7-point scale | “ | Nurses | 1673 |
| “ | Rospendowski et al., 2014 (23) | “ | Translation, validation | Brazil- Portuguese | “ | “ | “ | Nurses | 158 |
| “ | Pereira et al., 2015 (24) | “ | Translation, validation | Portugal- Portuguese | “ | 20 items/ 7-point scale | “ | Nurses | 358 |
| “ | Fajarini et al., 2021 (25) | “ | Translation, validation | Indonesia- Indonesian | “ | “ | “ | Nurses | 42 |
|  |  |  |  |  |  |  |  |  |  |
| The Evidence-based Practice Beliefs Scale (EBP beliefs) | Melnyk et al., 2008 (26) | EBP beliefs (self-efficacy) | Development, validation | USA- English | Ask, search, appraise, integrate, evaluate | 16 items/ 5-point scale | Unidimensional | Nurses | 394 |
| “ | Grønvik et al., 2016 (27) | “ | Translation, validation | Norway- Norwegian | “ | “ | Multidimensional/ 1. General knowledge and confidence concerning EBP, 2. Task specific beliefs in EBP, 3 and 4: Component 2 and 4 | Nurses, social workers | 112 |
| “ | Kerwien-Jacquier et al., 2020 (28) | “ | Translation, validation | Switzerland- German | “ | “ | Multidimensional/ value beliefs, implementation beliefs, knowledge beliefs, time and difficulty beliefs and effective evidence-based care beliefs | Nurses | 131 |
| “ | Thorsteinsson, 2012 (29) | “ | Translation, validation | Iceland- Icelandic | “ | “ | Unidimensional | Nurses | 540 |
| “ | Verloo et al., 2017 (30) | “ | Validation | Switzerland- French | “ | “ | Multidimensional/ knowledge beliefs, value beliefs, resource beliefs, and time and difficulty beliefs | Nurses, Physical therapists,  occupational therapists, others | 382 |
| “ | Van Giang et al., 2021 (12) | “ | Translation, validation | Vietnam/ Vietnamese | “ | “ | Multidimensional/ Value beliefs, Knowledge beliefs, resource beliefs | Nurses, midwifes | 353 |
|  |  |  |  |  |  |  |  |  |  |
| The EBP Beliefs Scale—Short  Version (EBP Beliefs- Short) | Melnyk et al., 2021 (31) | EBP beliefs (self-efficacy) | Adaption, validation | USA/ English | Integrate | 3 items/ 5-point scale | Unidimensional | Nurses | 498 |
|  |  |  |  |  |  |  |  |  |  |
| The Evidence-based Practice Implementation Scale (EBP implement) | Melnyk et al., 2008 (26) | EBP Implementation (behavior) | Development, validation | USA- English | Ask, search, appraise, integrate, evaluate | 18 items/ 5-point scale | Unidimensional | Nurses | 394 |
| “ | Kerwien-Jacquier et al., 2020 (28) | “ | Translation, validation | Switzerland- German | “ | 17 items/ 5-point scale | Multidimensional/ Use of EBP, scientific research and analysis, sharing knowledge of evidence, sharing and use of evidence-based guidelines, process of a practice change | Nurses | 131 |
| “ | Moore et al., 2018 (32) | “ | Validation | Norway- Norwegian | “ | 16 items/ 5-point scale | Multidimensional/ Literature Search and Appraisal, Knowledge Exchange, and Practice Evaluation | Physical Therapy  Occupational, Therapy, Nurse,  Nursing assistant,  Medical doctor, Psychologist, Social worker | 316 |
| “ | Verloo et al., 2017 (30) | “ | Validation | Switzerland- French | “ | 17 items/ 5-point scale | Multidimensional/ Seek and appraise scientific evidence and share evidence or data with colleagues or patients, and collect and evaluate outcomes and use evidence to change practice | Nurses, Physical therapists,  occupational therapists, others | 382 |
| The EBP Implementation Scale- Short version (EBP implement-Short) | Melnyk et al., 2021 (31) | EBP Implementation (behavior) | Adaption, validation | USA/ English | Ask, search, appraise, integrate, evaluate | 3 items/ 5-point scale | Unidimensional | Nurses | 498 |
| The EBP Implementation Scale—Ethiopian short version (Ethiopian EBP Implement) | Dessie et al., 2020 (33) | EBP Implementation (behavior) | Adaption, validation | Ethiopia/ Amharic | Search, integrate | 8 items/ 5-point scale | Multidimensional/ Search, Utilize | Nurses, midwifes, physicians | 405 |
|  |  |  |  |  |  |  |  |  |  |
| Measure of constructs relevant to evidence-based practice (Al Zoubi Questionnaire) | Al Zoubi et al., 2018 (34) | EBP attitudes, self-efficacy, resources, knowledge | Development, validation | Canada- English and French | Ask, search, appraise, integrate, evaluate | 40 items/ 5-point and 11-point scales | Multidimensional/ Attitudes, self-efficacy, resources, knowledge | Physical therapists,  occupational therapists | 128 |
|  |  |  |  |  |  |  |  |  |  |
| The Evidence-based Professional Practice Scale (EBPP-S) | Bernal & Ridriguez-Soto Ndel, 2010 (35) | EBP attitudes, behavior, and knowledge | Development, validation | Puerto Rico | ? | 17 items/ 5-point scale | Multidimensional/ attitudes, knowledge, behaviors | Mental health workers, psychologists | 151 |
|  |  |  |  |  |  |  |  |  |  |
| Evidence-Based Practice Questionnaire (EBP Jette) | Jette et al., 2003 (36) | EBP beliefs, attitudes, knowledge, behavior | Development, validation | USA- English | Ask, search, appraise, integrate, evaluate | 40 domain items/ 5-point scale and Yes/no | Dimensionality not stated or tested | Physical therapists | 54 |
| “ | Ferreira et al., 2020 (37) | “ | Translation, validation | Portugal- Portuguese | “ | “/ 4-pont-scale | Divided into sub-group, construct validity was not assessed since the instrument was intended  to measure item by item and not in factors. | Physical therapists | 72 |
|  |  |  |  |  |  |  |  |  |  |
| Questionnaire on the Use of and Attitudes Toward Evidence-Based Practice (EBP) and Guidelines in Physical Therapy (EBP Bernhardsson) | Bernhardsson & Larsson, 2013 (38) | EBP Attitudes, knowledge, behavior, prerequisites  and barriers | Development, validation | Sweden– Swedish | Ask, search, appraise, integrate, evaluate | 23 items/ 5-pont scale | Dimensionality not tested (“the instrument was intended to measure item by item and not in factors”) | Physical therapists | 42 |
|  |  |  |  |  |  |  |  |  |  |
| The Evidence-Based Practice Inventory (EBP inventory) | Kaper et al., 2015 (39) | EBP decision making, subjective norm, attitude, perceived behavior control, intention and behavior | Development, validation | The Netherlands/ International – English | Ask, search, appraise | 26 items/ 6-point scale | Multidimensional/ Decision making, subjective norm, attitude, perceived behavior control, intention and behavior | Medicine, Nursing, physiotherapy Sociology, Psychology | 537 (127) |
| “ | Braun et al., 2019 (40) | “ | Translation, validation | Germany- German | “ | “ | “ | Physical therapy, Occupational therapy, nurses, medical doctors, psychologists, midwifes, others | 889 |
|  |  |  |  |  |  |  |  |  |  |
| The evidence-based practice confidence (EPIC) scale | Salbach & Jaglal, 2011 (41) | EBP self-efficacy | Development, validation | Canada/ USA- English | Ask, search, appraise, integrate, evaluate | 11 items/ 11-point scale | Unidimensional | Physicians, nurses, physical therapists, occupational therapists, speech language pathologists and epidemiologist | 14 |
| “ | Salbach et al., 2013 (42) | “ | Validation | Canada- English | “ | “ | “ | Physical therapists |  |
| “ | Clyde et al., 2016 (43) | “ | Validation | Canada- English | “ | “ | “ | Occupational therapists |  |
|  |  |  |  |  |  |  |  |  |  |
| The modified practice attitudes scale (MPAS) | Borntrager et al., 2009 (44) | EBP attitudes | Development, validation | USA/ Hawaii- English | Integrate | 8 items/ 5-point scale | Unidimensional | Mental health workers | 55 |
| “ | Park et al., 2018 (45) | “ | Translation, validation | South Korea- Korean | “ | 5 items/ 5-point scale | “ | Mental health workers | Korea: 283  US: 189 |
|  |  |  |  |  |  |  |  |  |  |
| The Evidence-Based Practice Process Assessment Scale (EBPPAS) | Rubin & Parrish, 2010 (46) | EBP Knowledge, self-efficacy, attitudes, and behavior | Development, validation | USA- English | Ask, search, appraise, integrate, evaluate | 51 items/ 5-point scale | Multidimensional/ Familiarity, Attitudes, Feasibility, Intentions, currently engage | Social workers | 217 |
| “ | Rubin & Parrish, 2011 (47) | “ | Validation | USA- English | “ | 45 items/ 5-point scale | “ | Social workers, others | 511 |
|  |  |  |  |  |  |  |  |  |  |
| The Evidence-Based Practice Process Assessment Scale, short version (EBPPAS-s) | Parrish & Rubin 2011 (48) | EBP Knowledge, self-efficacy, attitudes, and behavior | Adaption, validation | USA- English | Ask, search, appraise, integrate, evaluate | 37 items/ 5-point scale | Multidimensional/ Familiarity, Attitudes, Feasibility, currently engage | Social workers, psychologists | 865 |
|  |  |  |  |  |  |  |  |  |  |
| Self-Efficacy in EBP (SE-EBP) scale | Chang & Crowe, 2011 (49) | EBP Self-efficacy | Development, validation | Australia- English | Ask, search, appraise, integrate, evaluate | 26 items/ 11-point scale | Multidimensional/ identifying the clinical problem, searching  for evidence, and implementing evidence into practice | Nurses, midwifes | 174 |
| “ | Oh et al., 2016 (50) | “ | Translation, validation | Korea- Korean | “ | 28 items/ 11-point scale | “ | Nurses | 212 |
|  |  |  |  |  |  |  |  |  |  |
| 17-item Evidence-Based Practice Self-efficacy (EBPSE) scale | Tucker et al., 2009 (51) | EBP Self-efficacy | Development, validation | USA- English | Ask, search, appraise, integrate, evaluate | 17 items/ scale of 1% to 100% | Unidimensional | Nurses | 93 |
|  |  |  |  |  |  |  |  |  |  |
| The EBP Capability Beliefs Scale | Wallin et al., 2012 (52) | EBP self-efficacy | Development, validation | Sweden- Swedish | Ask, search, appraise, integrate, evaluate | 6 items/ scale of 0 to 10 | Unidimensional | Nurses | 1256 |
|  |  |  |  |  |  |  |  |  |  |
| The Healthcare EBP Assessment Tool (HEAT) | Sleutel et al., 2015 (53) | EBP Behavior, self-efficacy, barriers | Development/ Revision, validation | USA- English | Ask, search, appraise, integrate | 23 items/ 5-point scale | Multidimensional/ EBP frequency, EBP Ability, EBP desire, EBP Barriers | Nurses | 2439 |
|  |  |  |  |  |  |  |  |  |  |
| The modified KAB scale (EBP-KABQ) | Shi et al., 2014 (54) | EBP knowledge, behavior, attitude | Development/ revision/modification, validation | International- English | Ask, search, appraise, integrate | 26 items/ 7 and 6-pont scales | Multidimensional/ knowledge, attitudes, behavior, outcomes/decisions | Nurses, Physical therapists, occupational therapists, Medical doctors, psychologists | 673 |
|  |  |  |  |  |  |  |  |  |  |
| The Quick EBP-VIK | Paul et al., 2016 (55) | EBP attitudes, knowledge, behavior | Development, validation | USA- English | Ask, search, appraise, integrate, evaluate | 25 items/ 5-point scale | Multidimensional/ Value, knowledge, implementation | Nurses, others, EBP experts | 34 |
| “ | Connor et al., 2017 (56) | “ | Validation | USA- English | “ | 19 items/ 5-point scale | “ | Nurses | 382 |
| “ | Zhou et al., 2019 (57) | “ | Translation, validation | China- Chinese | “ | 19 items/ 5-point scale | “ | Nurses | 402 |
|  |  |  |  |  |  |  |  |  |  |
| Health Sciences evidence-based practice questionnaire (HS-EBP) | Fernandez-Dominguez et al., 2016 (58) | EBP attitudes, behavior, barriers | Development validation | Spain- Spanish | Ask, search, appraise, integrate, evaluate | 73 items/ 10-point scale | Multidimensional/ Beliefs and attitudes, Results from scientific research,  Development of professional practice, Assessment of results,  Barriers– Facilitators | Physicians, physiotherapists, nurses and psychologists | 32 |
| “ | Fernandez-Dominguez et al., 2017 (59) | “ | Adaption, validation | Spain- Spanish | “ | 60 items/ 10-point scale | “ | Medical doctors, Nurses, Physical therapists, psychologists,  others | 869 |
|  |  |  |  |  |  |  |  |  |  |
| The Evidence-Based Practice Readiness Survey (EBPRS) | Thiel & Ghosh, 2008 (60) | EBP knowledge/ skills, behavior, attitudes, culture | Development | USA- English | Search, appraise integrate | 64 items/ 5-point scale | Multidimensional/ informational needs, EBP knowledge, EBP attitude, workplace culture | Nurses | 21 |
| “ | Patelarou et al., 2015 (61) | “ | Translation, adaption, validation | Greece- Greek | “ | “ | “ | Nurses | 477 |
|  |  |  |  |  |  |  |  |  |  |
| The evidence-based practice profile questionnaire (EBP2) | McEvoy et al., 2010 (62) | EBP attitudes, knowledge, self-efficacy, behavior | Development, validation | Australia- English | Ask, search, appraise, integrate, | 58 items/ 5-point scale | Multidimensional/ Relevance, Terminology, Confidence, practice, sympathy | Physiotherapy, Podiatry, Occupational Therapy, Human Movement, Medical Radiation, Nursing, Psychology, Commerce and Others | 526 |
| “ | Hu et al., 2020 (63) | “ | Translation, adaption, validation | China- Chinese | “ | 45 items/ 5-point scale | Multidimensional/ Basic Understanding, Intention,  Attitude, Sympathy, EBP-related terms, Clinical related terms, Practice,  Confidence | Nurses | 543 |
| “ | Panczyk et al., 2017 (64) | “ | Translation, validation | Poland- Polish | “ | “ | “ | Nurses, midwifes | 1362 |
| “ | Titlestad et al., 2017 (65) | “ | Translation, validation | Norway- Norwegian | “ | “ | “ | Nurses, physical therapists,  Occupational therapists, social workers, others | 149 |
| “ | Belowska et al., 2020 (66) | “ | Validation | Poland- Polish | “ | “ | “ | Nurses | 548 |
|  |  |  |  |  |  |  |  |  |  |
| The intention scale for providers-direct Items (ISP-D) | Burgess et al., 2017 (67) | EBP attitudes, behavior | Development, validation | USA- English | Integrate | 16 items/ 7-point scale | Multidimensional/ Attitudes, Subjective norms, Perceived behavioral control, Behavioral intention |  | 25 |
| “ | Mah et al., 2020 (68) | “ | Validation | Hawaii- English | “ | 14 items/ 7-point scale | “ | Mental health workers | 211 |
|  |  |  |  |  |  |  |  |  |  |
| Attitude towards Evidence-based Nursing Questionnaire (EBNAQ) | Ruzafa Martinez et al., 2011 (69) | EBP attitudes | Development, validation | Spain- Spanish | Search, integrate | 15 items/ 5-point scale | Multidimensional/ Beliefs and expectations towards EBN’, ‘Intention of conduct towards EBN’, ‘Feelings towards EBN’ | Nurses | 219 |
|  |  |  |  |  |  |  |  |  |  |
| Questionnaire on the current state of EBP engagement in Austria (EBP Diermayr) | Diermayr et al., 2015 (70) | EBP attitudes, behavior, self-efficacy | Development, validation | Austria- German | Search, appraise, integrate | 37 items/ 5-point scale | Multidimensional/ Attitude, Awareness of research evidence, Subjective norm, Training in scientific EBP skills, Perceived behaviour control, Availability of resources, Engagement in EBP activities, Barriers for EBP | Physical therapists | 588 |
|  |  |  |  |  |  |  |  |  |  |
| EBP Competency  Questionnaire, Professional version (EBP-COQ Prof©) | Ruzafa-Martinez et al., 2020 (71) | EBP Attitudes, knowledge, skills, utilization (behavior) | Development, validation | Spain- Spanish | Ask, search, appraise, integrate, evaluate | 35 items/ 5-point scale | Multidimensional/ Attitudes, knowledge, skills, utilization | Nurses | 579 |
| “ | Schetaki et al., 2022 (72) | “ | Translation, validation | Greece- Greek | “ | “ | “ | Nurses | 514 |
|  |  |  |  |  |  |  |  |  |  |
| EIDM competence measure | Belita et al., 2021 (73) | EIDM Knowledge, skills, attitudes/ beliefs, behavior | Develoment, validation | Canada/ English | Ask, search, appraise, integrate, evaluate | 40 items/ 7-point scale | Multidimensional/ Knowledge, skills, attitudes/ beliefs, behavior | Nurses, EIDM experts | 9, 11 |
|  |  |  |  |  |  |  |  |  |  |
| I-SABE | Ruano et al., 2022 (74) | EBP self-efficacy, behavior, attitudes, results | Development, validation | Brazil/ Brazilian Portugese | search, appraise, integrate | 31 items/ 7-point scale | Multidimensional/ EBP self-efficacy, behavior, attitudes, results | Medical doctors, pharmacist, nurses, dentists, physiotherapists, others | 217 |
|  |  |  |  |  |  |  |  |  |  |
| The Noor EBM questionnaire (Noor EBM) | Norhayati et al., 2022 (75) | EBM knowledge, attitude, and practice | Development, validation | Malaysia/ English | Ask, search, integrate | 45/ 5-point scale | Multidimensional/ EBM knowledge, attitude, and practice | Physicians | 90 |
|  |  |  |  |  |  |  |  |  |  |
| EBP-CBFRI | Abuadas et al., 2021 (76) | EBP Competence beliefs, barriers, facilitators, implementation | Development, validation | Saudi Arabia/ English | Ask, search, appraise, integrate, evaluate | 55/ 5-point scale | Multidimensional/ Competence beliefs, barriers, facilitators, implementation | Nurses | 612 |
|  |  |  |  |  |  |  |  |  |  |
| Abbreviations:  “ = Like the development study | | | | | | | | | |

1. Aarons GA. Mental health provider attitudes toward adoption of evidence-based practice: the Evidence-Based Practice Attitude Scale (EBPAS). Mental health services research. 2004;6(2):61-74.

2. Aarons GA, McDonald EJ, Sheehan AK, Walrath-Greene CM. Confirmatory factor analysis of the Evidence-Based Practice Attitude Scale in a geographically diverse sample of community mental health providers. Adm Policy Ment Health. 2007;34(5):465-9.

3. Aarons GA, Glisson C, Hoagwood K, Kelleher K, Landsverk J, Cafri G. Psychometric properties and U.S. National norms of the Evidence-Based Practice Attitude Scale (EBPAS). Psychol Assess. 2010;22(2):356-65.

4. Maessen K, van Vught A, Gerritsen DL, Lovink MH, Vermeulen H, Persoon A. Development and Validation of the Dutch EBPAS-ve and EBPQ-ve for Nursing Assistants and Nurses with a Vocational Education. Worldviews Evid Based Nurs. 2019;16(5):371-80.

5. Melas CD, Zampetakis LA, Dimopoulou A, Moustakis V. Evaluating the properties of the Evidence-Based Practice Attitude Scale (EBPAS) in health care. Psychol Assess. 2012;24(4):867-76.

6. Skavberg Roaldsen K, Halvarsson A. Reliability of the Swedish version of the Evidence-Based Practice Attitude Scale assessing physiotherapist's attitudes to implementation of evidence-based practice. PLoS ONE [Electronic Resource]. 2019;14(11):e0225467.

7. Egeland KM, Ruud T, Ogden T, Lindstrom JC, Heiervang KS. Psychometric properties of the Norwegian version of the Evidence-Based Practice Attitude Scale (EBPAS): to measure implementation readiness. Health research policy and systems. 2016;14(1):47.

8. Santesson A, Jarbin H, Holmberg R, Perrin S. Confirmatory factor analysis of the Evidence-Based Practice Attitude Scale in a large and representative sample of Child and Adolescent Mental Health practitioners: Is the use of a total scale score justified?: researchsquare.com; 2019.

9. Ashcraft RG, Foster SL, Lowery AE, Henggeler SW, Chapman JE, Rowland MD. Measuring practitioner attitudes toward evidence-based treatments: A validation study. Journal of Child & Adolescent Substance Abuse. 2011;20(2):166-83.

10. Baumann AA, Vazquez AL, Macchione AC, Lima A, Coelho AF, Juras M, et al. Translation and validation of the evidence-based practice attitude scale (EBPAS-15) to Brazilian Portuguese: Examining providers' perspective about evidence-based parent intervention. Children & Youth Services Review. 2022;136.

11. Ayhan Baser D, Agadayi E, Gonderen Cakmak S, Kahveci R. Adaptation of the evidence-based practices attitude scale-15 in Turkish family medicine residents. International Journal of Clinical Practice. 2021;75(8):e14354.

12. Van Giang N, Lin SY, Thai DH. A psychometric evaluation of the Vietnamese version of the Evidence-Based Practice Attitudes and Beliefs Scales. International Journal of Nursing Practice. 2021;27(6):e12896.

13. Aarons GA, Cafri G, Lugo L, Sawitzky A. Expanding the domains of attitudes towards evidence-based practice: the evidence based practice attitude scale-50. Adm Policy Ment Health. 2012;39(5):331-40.

14. Yildiz D, Fidanci BE, Acikel C, Kaygusuz N, Yildirim C. Evaluating the Properties of the Evidence-Based Practice Attitude Scale (EBPAS-50) in Nurses in Turkey. International Journal of Caring Sciences. 2018;11(2):768-75.

15. Rye M, Torres EM, Friborg O, Skre I, Aarons GA. The Evidence-based Practice Attitude Scale-36 (EBPAS-36): a brief and pragmatic measure of attitudes to evidence-based practice validated in US and Norwegian samples. Implementation science : IS. 2017;12(1):44.

16. Szota K, Thielemann JFB, Christiansen H, Rye M, Aarons GA, Barke A. Cross-cultural adaption and psychometric investigation of the German version of the Evidence Based Practice Attitude Scale (EBPAS-36D). Health Research Policy & Systems. 2021;19(1):90.

17. Upton D, Upton P. Development of an evidence-based practice questionnaire for nurses. J Adv Nurs. 2006;53(4):454-8.

18. Son Y-J, Song Y, Park S-Y, Kim J-I. A psychometric evaluation of the Korean version of the evidence-based practice questionnaire for nurses. Contemp Nurse. 2014;49(1):4-14.

19. Tomotaki A, Fukahori H, Sakai I, Kurokohchi K. The development and validation of the Evidence-Based Practice Questionnaire: Japanese version. Int J Nurs Pract. 2018;24(2):e12617.

20. Yang R, Guo JW, Beck SL, Jiang F, Tang S. Psychometric Properties of the Chinese Version of the Evidence-Based Practice Questionnaire for Nurses. J Nurs Meas. 2019;27(3):E117-E31.

21. Zaybak A, Gunes UY, Dikmen Y, Arslan GG. Cultural Validation of the Turkish Version of Evidence-Based Practice Questionnaire. International Journal of Caring Sciences. 2017;10(1):37-46.

22. Sese-Abad A, De Pedro-Gomez J, Bennasar-Veny M, Sastre P, Fernandez-Dominguez JC, Morales-Asencio JM. A multisample model validation of the evidence-based practice questionnaire. Res Nurs Health. 2014;37(5):437-46.

23. Rospendowiski K, Alexandre NMC, Cornello ME. Cultural adaptation to Brazil and psychometric performance of the "Evidence-Based Practice Questionnaire". Acta Paulista De Enfermagem. 2014;27(5):405-11.

24. Pereira RP, Guerra AC, Cardoso MJ, dos Santos AT, de Figueiredo Mdo C, Carneiro AC. Validation of the Portuguese version of the Evidence-Based Practice Questionnaire. Rev Lat Am Enfermagem. 2015;23(2):345-51.

25. Fajarini M, Rahayu S, Setiawan A. The indonesia version of evidence-based practice questionnaire (EBPQ): Translation and Reliability. The 6th Padjadjaran …. 2021.

26. Melnyk BM, Fineout-Overholt E, Mays MZ. The evidence-based practice beliefs and implementation scales: psychometric properties of two new instruments. Worldviews Evid Based Nurs. 2008;5(4):208-16.

27. Grønvik CKU, Ødegård A, Bjørkly S. Factor Analytical Examination of the Evidence-Based Practice Beliefs Scale: Indications of a Two-Factor Structure: scirp.org; 2016.

28. Kerwien-Jacquier E, Verloo H, Pereira F, Peter KA. Adaptation and validation of the evidence-based practice beliefs and implementation scales into German. Nursing Open. 2020:12.

29. Thorsteinsson HS. Translation and validation of two evidence-based nursing practice instruments. Int Nurs Rev. 2012;59(2):259-65.

30. Verloo H, Desmedt M, Morin D. Adaptation and validation of the Evidence-Based Practice Belief and Implementation scales for French-speaking Swiss nurses and allied healthcare providers. J Clin Nurs. 2017;26(17-18):2735-43.

31. Melnyk BM, Hsieh AP, Gallagher-Ford L, Thomas B, Guo J, Tan A, et al. Psychometric Properties of the Short Versions of the EBP Beliefs Scale, the EBP Implementation Scale, and the EBP Organizational Culture and Readiness Scale. Worldviews on Evidence-Based Nursing. 2021;18(4):243-50.

32. Moore JL, Friis S, Graham ID, Gundersen ET, Nordvik JE. Reported use of evidence in clinical practice: a survey of rehabilitation practices in Norway. BMC Health Serv Res. 2018;18(1):379.

33. Dessie G, Jara D, Alem G, Mulugeta H, Zewdu T, Wagnew F, et al. Evidence-Based Practice and Associated Factors Among Health Care Providers Working in Public Hospitals in Northwest Ethiopia During 2017. Current Therapeutic Research, Clinical & Experimental. 2020;93:100613.

34. Al Zoubi F, Mayo N, Rochette A, Thomas A. Applying modern measurement approaches to constructs relevant to evidence-based practice among Canadian physical and occupational therapists. Implementation Science. 2018;13(1):152.

35. Bernal G, Rodriguez-Soto Ndel C. Development and psychometric properties of the evidence-based professional practice scale (EBPP-S). P R Health Sci J. 2010;29(4):385-90.

36. Jette DU, Bacon K, Batty C, Carlson M, Ferland A, Hemingway RD, et al. Evidence-based practice: beliefs, attitudes, knowledge, and behaviors of physical therapists. Phys Ther. 2003;83(9):786-805.

37. Ferreira RM, Ferreira PL, Cavalheiro L, Duarte JA, Gonçalves RS. Evidence-based practice questionnaire for physical therapists: Portuguese translation, adaptation, validity, and reliability. Journal of Evidence-Based Healthcare. 2019;1(2):83-98.

38. Bernhardsson S, Larsson ME. Measuring evidence-based practice in physical therapy: translation, adaptation, further development, validation, and reliability test of a questionnaire. Phys Ther. 2013;93(6):819-32.

39. Kaper NM, Swennen MH, van Wijk AJ, Kalkman CJ, van Rheenen N, van der Graaf Y, et al. The "evidence-based practice inventory": reliability and validity was demonstrated for a novel instrument to identify barriers and facilitators for Evidence Based Practice in health care. J Clin Epidemiol. 2015;68(11):1261-9.

40. Braun T, Ehrenbrusthoff K, Bahns C, Happe L, Kopkow C. Cross-cultural adaptation, internal consistency, test-retest reliability and feasibility of the German version of the evidence-based practice inventory. BMC Health Serv Res. 2019;19(1):455.

41. Salbach NM, Jaglal SB. Creation and validation of the evidence-based practice confidence scale for health care professionals. J Eval Clin Pract. 2011;17(4):794-800.

42. Salbach NM, Jaglal SB, Williams JI. Reliability and validity of the evidence-based practice confidence (EPIC) scale. J Contin Educ Health Prof. 2013;33(1):33-40.

43. Clyde JH, Brooks D, Cameron JI, Salbach NM. Validation of the Evidence-Based Practice Confidence (EPIC) Scale With Occupational Therapists. Am J Occup Ther. 2016;70(2):7002280010p1-9.

44. Borntrager CF, Chorpita BF, Higa-McMillan C, Weisz JR. Provider attitudes toward evidence-based practices: are the concerns with the evidence or with the manuals? Psychiatr Serv. 2009;60(5):677-81.

45. Park H, Ebesutani CK, Chung KM, Stanick C. Cross-Cultural Validation of the Modified Practice Attitudes Scale: Initial Factor Analysis and a New Factor Model. Assessment. 2018;25(1):126-38.

46. Rubin A, Parrish DE. Development and validation of the Evidence-based Practice Process Assessment Scale: Preliminary findings. Research on Social Work Practice. 2010;20(6):629-40.

47. Rubin A, Parrish DE. Validation of the evidence-based practice Process Assessment Scale. Research on Social Work Practice. 2011;21(1):106-18.

48. Parrish DE, Rubin A. Validation of the Evidence-Based Practice Process Assessment Scale-Short Version. Research on Social Work Practice. 2011;21(2):200-11.

49. Chang AM, Crowe L. Validation of scales measuring self-efficacy and outcome expectancy in evidence-based practice. Worldviews Evid Based Nurs. 2011;8(2):106-15.

50. Oh EG, Yang YL, Sung JH, Park CG, Chang AM. Psychometric Properties of Korean Version of Self-Efficacy of Evidence-Based Practice Scale. Asian Nurs Res (Korean Soc Nurs Sci). 2016;10(3):207-12.

51. Tucker SJ, Olson ME, Frusti DK. Evidence-Based Practice Self-efficacy Scale Preliminary Reliability and Validity. Clin Nurse Spec. 2009;23(4):207-15.

52. Wallin L, Bostrom AM, Gustavsson JP. Capability beliefs regarding evidence-based practice are associated with application of EBP and research use: validation of a new measure. Worldviews Evid Based Nurs. 2012;9(3):139-48.

53. Sleutel MR, Barbosa-Leiker C, Wilson M. Psychometric Testing of the Health Care Evidence-Based Practice Assessment Tool. J Nurs Meas. 2015;23(3):485-98.

54. Shi Q, Chesworth BM, Law M, Haynes RB, MacDermid JC. A modified evidence-based practice- knowledge, attitudes, behaviour and decisions/outcomes questionnaire is valid across multiple professions involved in pain management. BMC Med Educ. 2014;14:263.

55. Paul F, Connor L, McCabe M, Ziniel S. The development and content validity testing of the Quick-EBP-VIK: a survey instrument measuring nurses' values, knowledge and implementation of evidence-based practice: researchgate.net; 2016.

56. Connor L, Paul F, McCabe M, Ziniel S. Measuring Nurses' Value, Implementation, and Knowledge of Evidence-Based Practice: Further Psychometric Testing of the Quick-EBP-VIK Survey. Worldviews Evid Based Nurs. 2017;14(1):10-21.

57. Zhou C, Wang Y, Wang S, Ou J, Wu Y. Translation, cultural adaptation, validation, and reliability study of the Quick-EBP-VIK instrument: Chinese version. J Eval Clin Pract. 2019;25(5):856-63.

58. Fernandez-Dominguez JC, Sese-Abad A, Morales-Asencio JM, Sastre-Fullana P, Pol-Castaneda S, de Pedro-Gomez JE. Content validity of a health science evidence-based practice questionnaire (HS-EBP) with a web-based modified Delphi approach. Int J Qual Health Care. 2016;28(6):764-73.

59. Fernandez-Dominguez JC, de Pedro-Gomez JE, Morales-Asencio JM, Bennasar-Veny M, Sastre-Fullana P, Sese-Abad A. Health Sciences-Evidence Based Practice questionnaire (HS-EBP) for measuring transprofessional evidence-based practice: Creation, development and psychometric validation. PLoS ONE [Electronic Resource]. 2017;12(5):e0177172.

60. Thiel L, Ghosh Y. Determining registered nurses' readiness for evidence-based practice. Worldviews Evid Based Nurs. 2008;5(4):182-92.

61. Patelarou AE, Dafermos V, Brokalaki H, Melas CD, Koukia E. The evidence-based practice readiness survey: a structural equation modeling approach for a Greek sample. International Journal of Evidence-Based Healthcare. 2015;13(2):77-86.

62. McEvoy MP, Williams MT, Olds TS. Development and psychometric testing of a trans-professional evidence-based practice profile questionnaire. Med Teach. 2010;32(9):e373-80.

63. Hu MY, Wu YN, McEvoy MP, Wang YF, Cong WL, Liu LP, et al. Development and validation of the Chinese version of the evidence-based practice profile questionnaire (EBP<sup>2</sup>Q). BMC Med Educ. 2020;20(1):280.

64. Panczyk M, Belowska J, Zarzeka A, Samolinski L, Zmuda-Trzebiatowska H, Gotlib J. Validation study of the Polish version of the Evidence-Based Practice Profile Questionnaire. BMC Med Educ. 2017;17(1):38.

65. Titlestad KB, Snibsoer AK, Stromme H, Nortvedt MW, Graverholt B, Espehaug B. Translation, cross-cultural adaption and measurement properties of the evidence-based practice profile. BMC Res Notes. 2017;10(1):44-.

66. Belowska J, Panczyk M, Zarzeka A, Iwanow L, Cieslak I, Gotlib J. Promoting evidence-based practice - perceived knowledge, behaviours and attitudes of Polish nurses: a cross-sectional validation study. International Journal of Occupational Safety & Ergonomics. 2020;26(2):397-405.

67. Burgess AM, Chang J, Nakamura BJ, Izmirian S, Okamura KH. Evidence-based practice implementation within a theory of planned behavior framework. The journal of behavioral health services & research. 2017;44(4):647-65.

68. Mah AC, Hill KA, Cicero DC, Nakamura BJ. A Psychometric Evaluation of the Intention Scale for Providers-Direct Items. J Behav Health Serv Res. 2020;47(2):245-63.

69. Ruzafa-Martinez M, Lopez-Iborra L, Madrigal-Torres M. Attitude towards Evidence-Based Nursing Questionnaire: development and psychometric testing in Spanish community nurses. J Eval Clin Pract. 2011;17(4):664-70.

70. Diermayr G, Schachner H, Eidenberger M, Lohkamp M, Salbach NM. Evidence-based practice in physical therapy in Austria: Current state and factors associated with EBP engagement. J Eval Clin Pract. 2015;21(6):1219-34.

71. Ruzafa-Martinez M, Fern, ez-Salazar S, Leal-Costa C, Ramos-Morcillo AJ. Questionnaire to Evaluate the Competency in Evidence-Based Practice of Registered Nurses (EBP-COQ Prof©): Development and Psychometric Validation. Worldviews on Evidence-Based Nursing. 2020;17(5):366-75.

72. Schetaki S, Patelarou E, Giakoumidakis K, Trivli A, Kleisiaris C, Patelarou A. Translation and Validation of the Greek Version of the Evidence-Based Practice Competency Questionnaire for Registered Nurses (EBP-COQ Prof©). Nursing Reports. 2022;12(4):693-707.

73. Belita E, Yost J, Squires JE, Ganann R, Dobbins M. Development and content validation of a measure to assess evidence-informed decision-making competence in public health nursing. PLoS ONE [Electronic Resource]. 2021;16(3):e0248330.

74. Ruano ASM, Motter FR, Lopes LC. Design and validity of an instrument to assess healthcare professionals' perceptions, behaviour, self-efficacy and attitudes towards evidence-based health practice: I-SABE. BMJ Open. 2022;12.

75. Norhayati MN, Nawi ZM. Validity and reliability of the Noor Evidence-Based Medicine Questionnaire: A cross-sectional study. PLoS ONE [Electronic Resource]. 2021;16(4):e0249660.

76. Abuadas MH, Albikawi ZF, Abuadas F. Development and Validation of Questionnaire Measuring Registered Nurses' Competencies, Beliefs, Facilitators, Barriers, and Implementation of Evidence-Based Practice (EBP-CBFRI). Journal of Nursing Measurement. 2021;13:13.
