# Supplementary material for: Instruments measuring evidence-based practice behavior, attitudes, and self-efficacy among healthcare professionals: a systematic review of measurement properties
Source: Implement Sci. 2023 Sep 13;18:42. doi: 10.1186/s13012-023-01301-3 (PMC10500884; doi:10.1186/s13012-023-01301-3)
Supplement: Supplementary file 4 — Additional file 4. Results of quality assessment and measurement properties of the individual studies. [file 13012_2023_1301_MOESM4_ESM.docx]

| **Additional file 4**  Results of quality assessment and measurement properties of the individual studies | | | | | | | | | | | | | |
| --- | --- | --- | --- | --- | --- | --- | --- | --- | --- | --- | --- | --- | --- |
|  | | **Box 3 Structural validity** | | | **Box 4 Internal consistency** | | | **Box 6 Reliability** | | | **Box 7 Measurement error** | | |
| **Reference** | **Instrument** | **n** | **Meth qual** | **Result (rating)** | **n** | **Meth qual** | **Result (rating) *** | **n** | **Meth qual** | **Result (rating) *** | **n** | **Meth qual** | **Result (rating)** |
|  |  |  |  |  |  |  |  |  |  |  |  |  |  |
| Aarons, 2004 (1) | **EBPAS** | 322 | VG | EFA and CFA: SRMR= 0.077 **(+)** | 322 | VG | α = 0.90 **(+)**, 0.80 **(+)**, 0.78 **(+)**, 0.59 **(-)** | --- | --- | --- | --- | --- | --- |
| Aarons et al., 2007 (2) | EBPAS | 221 | VG | CFA: SRMR= 0.07 **(+)** | 221 | VG | α = 0.93 **(+)**, 0.74 **(+)**, 0.81 **(+)**, 0.66 **(-)** | --- | --- | --- | --- | --- | --- |
| Aarons et al., 2010 (3) | EBPAS | 1089 | VG | CFA: SRMR= 0.058 **(+)** | 1089 | VG | α = 0.93 **(+)**, 0.74 **(+)**, 0.81 **(+)**,0.66 **(-)** | --- | --- | --- | --- | --- | --- |
| Maessen et al., 2019 (4) | EBPAS | 273 | A | EFA Factor loadings > 0.3, cross-loading <10%, and tot variance explained = 63.2% **(+)** | 273 | VG | α = 0.90 **(+)**, 0.78 **(+)**, 0.73 **(+)**, 0.55 **(-)** | 80 | A | ICC= 0.55 **(-)**, 0.40 **(-)**, 0.64 **(-)**, 0.48 **(-)** | --- | --- | --- |
| Melas et al., 2012 (5) | EBPAS | 534 | VG | CFA: SRMR= 0.099 **(-)** | --- | --- | --- | --- | --- | --- | --- | --- | --- |
| Skavberg Roaldsen & Halvarsson, 2019 (6) | EBPAS | --- | --- | --- | 55 | VG | α = 0.89 **(+)**, 0.72 **(+)**, 0.83 **(+)**, 0.85 **(+)** | 55 | VG | ICC= 0.80 **(+)**, 0.56 **(-)**, 0.71 **(+)**, 0.74 **(+)** | 55 | VG | LoA −1.028–0.944 < SRD 0.96 **(+)**,  LoA −1.183–0.965 < SRD 1.066 **(+)**,  LoA −0.952–0.824 < SRD 0.827 **(+)**,  LoA −0.960–0.896 < SRD 0.902 **(+)** |
| Egeland et al., 2016 (7) | EBPAS | 294 | VG | CFA: SRMR= 0.081 **(-)** | 294 | VG | α = 0.88 **(+)**, 0.74 **(+)**, 0.82 **(+)**, 0.64 **(-)** | --- | --- | --- | --- | --- | --- |
| Santesson et al., 2019 (8) | EBPAS | 565 | VG | CFA: SRMR=0.061 **(+)** | 565 | VG | α = 0.88 **(+)**, 0.74 **(+)**, 0.76 **(+)**, 0.60 **(-)** | --- | --- | --- | --- | --- | --- |
| Ashcraft et al., 2011 (9) | EBPAS | 543 | VG | CFA: SRMR=0.06 **(+)** | 543 | VG | α = 0.94 **(+)**, 0.74 **(+)**, 0.82 **(+)**, 0.51 **(-)** | --- | --- | --- | --- | --- | --- |
| Baumann et al., 2022 (10) | EBPAS | 362 | VG | CFA: SRMR= 0.069 **(+)** | 362 | VG | α = 0.86 **(+)**, 0.74 **(+)**, 0.77 **(+)**, 0.60 **(-)** | --- | --- | --- | --- | --- | --- |
| Ayhan Baser et al., 2021 (11) | EBPAS | 151 | VG | CFA:  RMSEA = 0.062 **(-)** | 151 | I | α = 0.828 on total scale **(?)** | --- | --- | --- | --- | --- | --- |
| Van Giang et al., 2021 (12) | EBPAS | 353 | VG | CFA:  SRMR = 0.064 **(+)** | 353 | VG | α = 0.86 **(+),** 0.76 **(+**), 0.81 **(+),** 0.67 **(-)** | --- | --- | --- | --- | --- | --- |
|  |  |  |  |  |  |  |  |  |  |  |  |  |  |
| Aarons et al., 2012 (13) | **EBPAS-50** | 420 | A | EFA: (eight factors) Factor loadings > 0.3, cross-loading <10%, and tot variance explained > 50% **(+)** | 420 | VG | α on the 8 new subscales range = 0.77-0.92 **(+)** | --- | --- | --- | --- | --- | --- |
| Yildiz et al., 2018 (14) | EBPAS-50 | 250 | D | CFA: No model fit indices reported **(?)** | 250 | I | α = 0.783 on total scale **(?)** | 40 | D | Pearson correlation total scale = 0.344. No ICC calculated **(?)** | --- | --- | --- |
|  |  |  |  |  |  |  |  |  |  |  |  |  |  |
| Rye et al., 2017 (15) | **EBPAS-36** | 838 | VG | CFA: RMSEA= 0.052 **(+)** | 838 | VG | Appeal, Fit, Balance and Divergence subscales α = 0.61, 0.62, 0.64 and 0.68. **(-)**. α = >0.70 on rest of subscales **(+)** | --- | --- | --- | --- | --- | --- |
| Szota et al., 2021 (16) | EBPAS-36 | 278 | VG | CFA: RMSEA= .064 **(-)** | 599 | VG | Appeal, Fit, Balance and Divergence subscales α = 0.69, 0.68, 0.65 and 0.66. **(-)**. α = >0.75 on rest of subscales **(+)** | --- | --- | --- | --- | --- | --- |
|  |  |  |  |  |  |  |  |  |  |  |  |  |  |
| Upton & Upton, 2006 (17) | **EBPQ** | 500 | A | EFA: Factor loadings >0.3, cross-loading <10%, and tot variance explained = 61.77% **(+)** | 500 | VG | α = 0.79 **(+)**, 0.85 **(+)**, 0.91 **(+)** | --- | --- | --- | --- | --- | --- |
| Son et al., 2014 (18) | EBPQ | 397 | VG | CFA: SRMR= 0.06 **(+)** | 404 | VG | α = 0.819 **(+)**, 0.917 **(+)**, 0.954 **(+)** | --- | --- | --- | --- | --- | --- |
| Tomotaki et al., 2018 (19) | EBPQ | 501 | VG | CFA: SRMR=0.102 **(-)** | 501 | VG | α = 0.69 **(-)**, 0.93 **(+)**, 0.91 **(+)**, 0.90 **(+)** | 108 | D | ICC= 0.44 **(-)**, 0.78 **(+)**, 0.70 **(+)**, 0.75 **(+)** | --- | --- | --- |
| Yang et al., 2019 (20) | EBPQ | 810 | A | EFA: Factor loadings >0.3, cross-loading <10%, and tot variance explained = 57.03 **(+)** | 810 | VG | α = 0.83 **(+)**, 0.86 **(+)**, 0.94 **(+)** | --- | --- | --- | --- | --- | --- |
| Zaybak et al., 2017 (21) | EBPQ | 123 | A | CFA: SRMR= 0.051 **(+)** | 123 | VG | α = 0.80 **(+)**, 0.93 **(+)**, 0.94 **(+)** | 30 | D | Pearson correlation= 0.94 **(?)**, 0.96 **(?)**, 0.97 **(?)**. No ICC calculated **(?)** | --- | --- | --- |
| Sese-Abad et al., 2014 (22) | EBPQ | 1673 | VG | CFA: SRMR= 0.06 **(+)** | 1673 | VG | α = 0.71 **(+)**, 0.86 **(+)**, 0.90 **(+)** | --- | --- | --- | --- | --- | --- |
| Rospendowski et al., 2014 (23) | EBPQ | --- | --- | --- | 158 | VG | α = 0.68 **(-)**, 0.86 **(+)**, 0.92 **(+)** | 50 | D | ICC= 0.86 **(+)**, 0.84 **(+)**, 0.86 **(+)** | --- | --- | --- |
| Pereira et al., 2015 (24) | EBPQ | 358 | VG | CFA: CFI = 0.908 **(-)** | 358 | VG | α = 0.75 **(+)**, 0.74 **(+)**, 0.95 **(+)** | --- | --- | --- | --- | --- | --- |
| Fajarini et al., 2021 (25) | EBPQ | -- | --- | --- | 42 | VG | α = 0.80 **(+)**, 0.92 **(+)**, 0.96 **(+)** | --- | --- | --- | --- | --- | --- |
|  |  |  |  |  |  |  |  |  |  |  |  |  |  |
| Melnyk et al., 2008 (26) | **EBP Beliefs** | 333 | D | EFA (Single factor): Factor loadings >0.35, Eigenvalue >1 **(+)** | 333 | VG | α =0.90 **(+)** | --- | --- | --- | --- | --- | --- |
| Grønvik et al., 2016 (27) | EBP Beliefs | 112 | A | EFA (four factors): Factor loading > 0.52, tot variance explained = 55%, cross-loading not reported **(?)** | 112 | VG | α = 0.72 **(+)**, 0.70 **(+),** 0.53 **(-)**, 0.54 **(-)** | --- | --- | --- | --- | --- | --- |
| Kerwien-Jacquier et al., 2020 (28) | EBP Beliefs | 131 | A | EFA (four factors):  Factor loading > 0.458, tot variance explained = 67.47%, cross-loading >10% **(-)** | 131 | VG | α =0.814 **(+)**, 0.770 **(+)**, 0.757 **(+**), 0.653 **(-)** | --- | --- | --- | --- | --- | --- |
| Thorsteinsson, 2012 (29) | EBP Beliefs | 471 | D | EFA (single factor):  Factor loading >0.45, Eigenvalue >1 **(+)** | 471 | VG | α = 0.86 **(+)** | --- | --- | --- | --- | --- | --- |
| Verloo et al., 2017 (30) | EBP Beliefs | 382 | A | EFA (four factors):  Factor loading >0.446, Eigenvalue >1, Cross-loading >10 % **(-)** | 382 | VG | α = 0.829 **(+)**, 0.872 **(+)**, 0.571  **(-)**, 0.655 **(-)** | --- | --- | --- | --- | --- | --- |
| Van Giang et al., 2021 (12) | EBP Beliefs | 353 | VG | CFA (three factor): SRMR = 0.056 **(+)** | 353 | VG | α = 0.83 (**+)**, 0.68 **(-)**, 0.64 **(-)** | --- | --- | --- | --- | --- | --- |
|  |  |  |  |  |  |  |  |  |  |  |  |  |  |
| Melnyk et al., 2021 (31) | **EBP Beliefs- Short** | 498 | D | EFA (single factor): Factor loading > 0.70, Eigenvalue = 2.25 **(+)** | 498 | VG | α = 0.89 **(+)** | --- | --- | --- | --- | --- | --- |
|  |  |  |  |  |  |  |  |  |  |  |  |  |  |
| Melnyk et al., 2008 (26) | **EBP Implement** | 319 | D | EFA (single factor): Factor loading >0.60, Eigenvalue >1 **(+)** | 319 | VG | α = 0.96 **(+)** | --- | --- | --- | --- | --- | --- |
| Kerwien-Jacquier et al., 2020 (28) | EBP Implement | 131 | A | EFA (five factors): Factor loading > 0.468, total variance explained = 68.79 %, cross-loading > 10%  **(-)** | 131 | VG | α =0.841 **(+)**, 0.865 **(+)**, 0.631 **(-)**, 0.639 **(-)**, 0.261 **(-)** | --- | --- | --- | --- | --- | --- |
| Moore et al., 2018 (32) | EBP Implement | 316 | D | EFA (three factors): factor loading >0.30, eigenvalues, total explained variance and cross-loading not reported **(?)** | 316 | VG | α =0.80 **(+),** 0.83 **(+)**, 0.74 **(+)** | --- | --- | --- | --- | --- | --- |
| Verloo et al., 2017 (30) | EBP Implement | 382 | A | EFA (Two factors): factor loading > 0.577, eigenvalues > 1, Cross-loading > 10% **(-)** | 382 | I | α = 0.954 (total scale) **(?)** | --- | --- | --- | --- | --- | --- |
| Melnyk et al., 2021 (31) | **EBP Implement- short** | 498 | D | EFA (single factor): Factor loading > 0.85, Eigenvalue = 2.46 **(+)** | 498 | VG | α = 0.81 **(+)** | --- | --- | --- | --- | --- | --- |
|  |  |  |  |  |  |  |  |  |  |  |  |  |  |
| Dessie et al., 2020 (33) | **Ethiopian EBP Implement** | 405 | A | EFA (two factors): Factor loadings > 0.40, Eigenvalues >1, cross-loading, no cross loadings **(+)** | 405 | I | Cronbachs alpha not reported for each subscale. total scale= 0.83 **(?)** | --- | --- | --- | --- | --- | --- |
|  |  |  |  |  |  |  |  |  |  |  |  |  |  |
| Al Zoubi et al., 2018 (34) | **Al Zoubi Questionnaire** | 128 | A | IRT/ Rasch: Model fit:  x2 >0.01 on three out of four subscales, . Local independence: items correlating >0.3 led to removal, Monotonicity and unidimensionality not reported **(?)** | 128 | VG | Person separation index **= 0.63, 0.80, 0.81, 0.86 (?)** | --- | --- | --- | --- | --- | --- |
|  |  |  |  |  |  |  |  |  |  |  |  |  |  |
| Bernal & Rodriguez-Soto Ndel, 2010 (35) | **EBPP-S** | 86 | D | CFA: CFI= .96, and RMSEA = .06 **(+)** | 86 | VG | α = 0.89 **(+)**, 0.90 **(+)**, 0.83 **(+)** | --- | --- | --- | --- | --- | --- |
|  |  |  |  |  |  |  |  |  |  |  |  |  |  |
| Jette et al., 2003 (36) | **EBP (Jette)** | --- | --- | --- | --- | --- | --- | 54 | A | ICC ranged from 0.37 to 0.90 (50% > 0.70). ICC per item not reported **(?)** | --- | --- | --- |
| Ferreira et al., 2020 (37) | **EBP (Jette)** | --- | --- | --- | 72 | D | Not report results due to no defined constructs | 72 | I | percentage of agreement. No ICC or Pearson or Spearman correlations calculated **(?)** | --- | --- | --- |
| Bernhardsson & Larsson, 2013 (38) | **EBP (Bernhardsson)** | --- | --- | --- | --- | --- | --- | 42 | I | percentage of agreement. No ICC or Pearson or Spearman correlations calculated **(?)** | --- | --- | --- |
|  |  |  |  |  |  |  |  |  |  |  |  |  |  |
| Kaper et al., 2015 (39) | **EBP inventory** | 127 | I | EFA: Factor loadings >0.30, cross-loading and tot variance explained/ eigenvalue not reported **(?)** | 127 | VG | α = > 0.7 on all subscales **(+)** except “Decision making” subscale (α = 0.6) **(-)** | 93 | A | ICC= 0.71 **(+)**, 0.63 **(-)**, 0.53 **(-)**, 0.83 **(+)**, 0.76 **(+)** | --- | --- | --- |
| Braun et al., 2019 (40) | EBP inventory | --- | --- | --- | 889 | VG | α = 0.64 **(-)**, 0.86 **(+)**, 0.90 **(+)**, 0.89 **(+)**, 0.83 **(+)** | 344 | VG | ICC= 0.78 **(+)**, 0.86 **(+)**, 0.82 **(+)**, 0.80 **(+)**, 0.76 **(+)** | 344 | VG | SEM calculated. MIC not defined **(?)** |
|  |  |  |  |  |  |  |  |  |  |  |  |  |  |
| Salbach et al., 2013 (41) | **EPIC** | 275 | A | EFA: All items loaded into one single factor >0.4, Tot explained variance = 71%, <10% cross-loading **(+)** | 275 | VG | α = 0.89 **(+)** | 192 | A | ICC= 0.89 **(+)** | 192 | A | LoA calculated. MIC not defined **(?)** |
| Clyde et al., 2016 (42) | EPIC | --- | --- | --- | --- | --- | --- | 79 | D | ICC= 0.92 **(+)** | 79 | D | MDC calculated. MIC not defined **(?)** |
|  |  |  |  |  |  |  |  |  |  |  |  |  |  |
| Borntrager et al., 2009 (43) | **MPAS** | --- | --- | --- | 55 | VG | α = 0.80 **(+)** | --- | --- | --- | --- | --- | --- |
| Park et al., 2018 (44) | MPAS | US: 189  Korea:  283 | VG | US: CFA: RMSEA= 0.030, CFI 0.998 **(+)**  Korea: CFA: RMSEA= <0.05, CFI >0.95 **(+)** | US: 189  Korea:  283 | VG | US: α = 0.78 **(+)**  Korea: α = 0.65 **(-)** | --- | --- | --- | --- | --- | --- |
|  |  |  |  |  |  |  |  |  |  |  |  |  |  |
| Rubin & Parrish, 2010 (45) | **EBPPAS** | 217 | I | EFA: Factor loadings= 0.68.0.88, cross-loading <10%, and eigenvalues >1 **(+)** | 217 | VG | α = 0.92 **(+)**, 0.90 **(+)**, 0.57 **(-)**. 0.80 **(+)**, 0.87 **(+)** | --- | --- | --- | --- | --- | --- |
| Rubin & Parrish, 2011 (46) | EBPPAS | 511 | VG | CFA: CFI= 0.90 **(-)** | 511 | VG | α = 0.91 **(+)**, 0.83 **(+)**, 0.63 **(-)**, 0.86 **(+)**, 0.86 **(+)** | --- | --- | --- | --- | --- | --- |
|  |  |  |  |  |  |  |  |  |  |  |  |  |  |
| Parrish & Rubin 2011 (47) | **EBPPAS-s** | 865 | VG | CFA: CFI= 0.90, RMSEA= 0.06 **(-)** | 865 | VG | α = 0.93 **(+)**, 0.92 **(+)**, 0.74 **(+)**, 0.91 **(+)** | --- | --- | --- | --- | --- | --- |
|  |  |  |  |  |  |  |  |  |  |  |  |  |  |
| Chang & Crowe, 2011 (48) | **SE-EBP** | 174 | A | EFA: Factor loadings >0.30, tot explained variance = 73.01%, all items loaded into one of the three factors **(+)** | 174 | VG | α = 0.91 **(+)**, 0.96 **(+)**, 0.96 **(+)** | --- | --- | --- | --- | --- | --- |
| Oh et al., 2016 (49) | SE-EBP | 212 | VG | CFA: CFI = 0.91 **(-)** | 212 | VG | α = 0.89 **(+)**, 0.90 **(+)**, 0.93 **(+)** | --- | --- | --- | --- | --- | --- |
|  |  |  |  |  |  |  |  |  |  |  |  |  |  |
| Tucker et al., 2009 (50) | **EBPSE** | --- | --- | --- | 93 | VG | α = 0.95 **(+)** | --- | --- | --- | --- | --- | --- |
|  |  |  |  |  |  |  |  |  |  |  |  |  |  |
| Wallin et al., 2012 (51) | **EBP Capability Beliefs** | 545 | VG | IRT/ Rasch: Model fit:  x2= 42,71. Local independence: Two cross-loadings (0.23 and 0.30). Monotonicity and unidimensionality not reported **(?)** | 545 | VG | person separation index = 0.92 **(?)** | --- | --- | --- | --- | --- | --- |
|  |  |  |  |  |  |  |  |  |  |  |  |  |  |
| Sleutel et al., 2015 (52) | **HEAT** | 1219 | VG | CFA: SRMR= 0 .063 **(+)** | 2439 | VG | α = 0.82 **(+)**, 0.89 **(+)**, 0.92 **(+)**, 0.80 **(+)** | --- | --- | --- | --- | --- | --- |
|  |  |  |  |  |  |  |  |  |  |  |  |  |  |
| Shi et al., 2014 (53) | **EBP-KABQ** | 673 | VG | CFA: CFI = 0.89, TLI = 0.86, RMSEA = 0.06 **(-)** | 673 | VG | α = 0.66 **(-)**, 0.75 **(+**), 0.77 **(+),** 0.83 **(+)** | --- | --- | --- | --- | --- | --- |
|  |  |  |  |  |  |  |  |  |  |  |  |  |  |
| Connor et al., 2017 (54) | **Quick EBP-VIK** | 382 | D | EFA: Factor loadings >0.30, cross-loading and tot variance explained/ eigenvalue not reported **(?)** | 382 | VG | α = 0.78 **(+)**, 0.93 **(+**), 0.66 **(-)** | 131 | A | ICC= 0.51 **(-)**, 0.70 **(+)**, 0.63 **(+)** | --- | --- | --- |
| Zhou et al., 2019 (55) | Quick EBP-VIK | 209 | VG | CFA: CFI 0.957 **(+)** | 402 | VG | α = 0.89 **(+)**, 0.92 **(+)**, 0.91 **(+)** | 20 | A | ICC= 0.57 **(-)**, 0.88 **(+)**, 0.84 **(+)** | --- | --- | --- |
|  |  |  |  |  |  |  |  |  |  |  |  |  |  |
| Fernandez-Dominguez et al., 2017 (56) | **HS-EBP** | 869 | VG | CFA: CFI =0.99, RMSEA = 0.047, SRMR = 0.067 **(+)** | 869 | VG | α = 0.93 **(+)**, 0.96 **(+**), 0.84 **(+)**, 0.94 **(+)**, 0.91 **(+)** | --- | --- | --- | --- | --- | --- |
|  |  |  |  |  |  |  |  |  |  |  |  |  |  |
| Patelarou et al., 2015 (57) | **EBPRS** | 477 | VG | CFA: CFI= 0.96, TLI= 0.96, SRMR= 0.058 **(+)** | 477 | VG | α = 0.85 **(+)**, 0.78 **(+)**, 0.87 **(+)**, 0.84 **(+)** | --- | --- | --- | --- | --- | --- |
|  |  |  |  |  |  |  |  |  |  |  |  |  |  |
| McEvoy et al., 2010 (58) | **EBP2** | 526 | A | EFA (five factors): Eigenvalue >1, cross-loading and factor lading not reported **(?)** | 106 | VG | α =0.94 **(+)**, 0.94 **(+**), 0.93 **(+)**, 0.85 **(+)**, 0.76 **(+)** | 106 | A | ICC= 0.92 **(+),** 0.94 **(+)**, 0.83 **(+)**, 0.83 **(+)**,0.77 **(+)** | --- | --- | --- |
| Hu et al., 2020 (59) | EBP2 | 250 | VG | CFA (8 factors): CFI 0.88 **(-)** | 543 | VG | α =0.91 **(+)**, 0.95 **(+)**, 0.90 **(+)**, 0.85 **(+)**, 0.85 (**+)**, 0.87 **(+)**, 0.92 **(+)**, 0.95 **(+)** | 22 | A | ICC= 0.84**(+)**, 0.80 **(+)**,0.91 **(+)**, 0.75 **(+)**, 0.90 **(+**),0.90 **(+)**, 0.84 **(+)**, 0.96 **(+)** | --- | --- | --- |
| Panczyk et al., 2017 (60) | EBP2 | 1362 | A | EFA (five factors): tot variance explained 63%, factor loading >0.3, cross-loading <10% **(+)** | 1362 | VG | α =0.94 **(+)**, 0.97 **(+)**, 0.94 **(+)**, 0.92 **(+)**, 0.80 **(+)** | 160 | A | ICC= 0.94 **(+)**, 0.97 **(+)**, 0.95 **(+)**, 0.92 **(+)**, 0.68 **(-)** | --- | --- | --- |
| Titlestad et al., 2017 (61) | EBP2 | 149 | VG | CFA (five factors): CFI: 0.68 **(-)** | 149 | VG | α = 0.91 **(+),** 0.94 **(+)**, 0.94 **(+)**, 0.90 (**+)**, 0.66 **(-)** | 53 | A | ICC= 0.69 **(-)**, 0.79 **(+)**, 0.76 **(+),** 0.45 **(-)**, 0.47 **(-)** | 53 | A | SEM calculated. MIC not defined **(?)** |
| Belowska et al., 2020 (62) | EBP2 | --- | --- | --- | 548 | VG | α = 0.94 **(+**), 0.98 **(+)**, 0.94 **(+)**, 0.92 **(+)**, 0.80 **(+)** | --- | --- | --- | --- | --- | --- |
|  |  |  |  |  |  |  |  |  |  |  |  |  |  |
| Mah et al., 2020 (63) | **ISP-D** | 211 | VG | CFA (four factors): SRMR= 0.075 **(+)** | 211 | VG | α = 0.75 **(+)**, 0.72 **(+)**, 0.63 **(-)**, 0.84 **(+)** | --- | --- | --- | --- | --- | --- |
|  |  |  |  |  |  |  |  |  |  |  |  |  |  |
| Ruzafa Martinez et al., 2011 (64) | **EBNAQ** | 219 | A | EFA (three factors): tot variance explained 54.7%, factor loading > 0.3, cross-loading < 10% **(+)** | 219 | VG | α = 0.86 **(+)**, 0.63 **(+)**, 0.70 **(+)** | --- | --- | --- | --- | --- | --- |
|  |  |  |  |  |  |  |  |  |  |  |  |  |  |
| Diermayr et al., 2015 (65) | **(EBP Diermayr)** | --- | --- | --- | --- | --- | --- | 32 | D | Mean ICC 0.67 (0.40-0.89) **(-)** | --- | --- | --- |
|  |  |  |  |  |  |  |  |  |  |  |  |  |  |
| Ruzafa-Martinez et al., 2020 (66) | **EBP-COQ Prof** | 579 | VG | CFA (Four factors): CFI = 0.932 **(-)** | 579 | VG | α = 0.888 **(+)**, 0.948 **(+)**, 0.817 **(+**), 0.840 **(+)** | 18 | A | ICC= 0.840 **(+)**, 0.966**(+)**, 0.815)**(+)**, 0.876 **(+)** | --- | --- | --- |
| Schetaki et al., 2022 (67) | EBP-COQ Prof | 414 | VG | CFA (Four factors): CFI = 0.82 **(-)** | 414 | VG | Cronbach’s alpha ranging from 0.918 to 0.952 **(+)** | 20 | A | Only for total scale. ICC= 0.994 (+) **??** | --- | --- | ---- |
|  |  |  |  |  |  |  |  |  |  |  |  |  |  |
| Ruano et al., 2022 (68) | **I-SABE** | 217 | A | EFA (four factors): tot variance explained 52.6%, factor loading > 0.3, cross-loading > 10% **(-)** | 217 | VG | α = 0.76 **(+)**, 0.30 **(-)**, 0.64 **(-),** 0.84 (**+)** | --- | --- | --- | --- | --- | --- |
|  |  |  |  |  |  |  |  |  |  |  |  |  |  |
| Norhayati et al., 2022(69) | **Noor EBM** | 90 | A | EFA: Attitude scale: five factors. Factor loading= 0.44-0.92, tot variance= 66.29%. Cross-loading not reported **(?)**  Practice scale: Two-factor. Factor loading= 0.43-0.99, tot variance= 55.39. Cross-loading not reported **(?)** | 90 | I | α (on total scales): Attitudes scale= 0.81 **(?),** Practice scale= 0.84 **(?)** | --- | --- | --- | --- | --- | --- |
|  |  |  |  |  |  |  |  |  |  |  |  |  |  |
| Abuadas et al., 2021(70) | **EBP-CBFRI** | 612 | VG | CFA (five factors): RMSA= 0.05 **(+)** | 612 | VG | Cronbach’s alpha ranging from 0.92 to 0.95 **(+)** | --- | --- | --- | --- | --- | --- |
|  |  |  |  |  |  |  |  |  |  |  |  |  |  |
| Abbreviations:  --- = no info available  Meth qual: results of quality assessment  VG= very good; A= adequate; D= doubtful; I= inadequate  Rating of results: (+) = sufficient result; (-) = insufficient result; (?) = indeterminate result  Model fit: CFI; Comparative fit index; RMSEA= root mean square error of approximation; SRMR= standardized square residual (71)  EFA= exploratory factor analysis (72); CFA= confirmatory factor analysis  α = Cronbach’s alpha  ICC= Intraclass correlation coefficient  SEM= Standard error of measurement; LoA= limits of agreement; SDC/MDC= smallest/minimal detectable change; MIC = minimal important change  *= Results on separate subscales are reported in the same order as the subscales are listed under “subscales” in table 1, unless otherwise stated | | | | | | | | | | | | | |

1. Aarons GA. Mental health provider attitudes toward adoption of evidence-based practice: the Evidence-Based Practice Attitude Scale (EBPAS). Mental health services research. 2004;6(2):61-74.

2. Aarons GA, McDonald EJ, Sheehan AK, Walrath-Greene CM. Confirmatory factor analysis of the Evidence-Based Practice Attitude Scale in a geographically diverse sample of community mental health providers. Adm Policy Ment Health. 2007;34(5):465-9.

3. Aarons GA, Glisson C, Hoagwood K, Kelleher K, Landsverk J, Cafri G. Psychometric properties and U.S. National norms of the Evidence-Based Practice Attitude Scale (EBPAS). Psychol Assess. 2010;22(2):356-65.

4. Maessen K, van Vught A, Gerritsen DL, Lovink MH, Vermeulen H, Persoon A. Development and Validation of the Dutch EBPAS-ve and EBPQ-ve for Nursing Assistants and Nurses with a Vocational Education. Worldviews Evid Based Nurs. 2019;16(5):371-80.

5. Melas CD, Zampetakis LA, Dimopoulou A, Moustakis V. Evaluating the properties of the Evidence-Based Practice Attitude Scale (EBPAS) in health care. Psychol Assess. 2012;24(4):867-76.

6. Skavberg Roaldsen K, Halvarsson A. Reliability of the Swedish version of the Evidence-Based Practice Attitude Scale assessing physiotherapist's attitudes to implementation of evidence-based practice. PLoS ONE [Electronic Resource]. 2019;14(11):e0225467.

7. Egeland KM, Ruud T, Ogden T, Lindstrom JC, Heiervang KS. Psychometric properties of the Norwegian version of the Evidence-Based Practice Attitude Scale (EBPAS): to measure implementation readiness. Health research policy and systems. 2016;14(1):47.

8. Santesson A, Jarbin H, Holmberg R, Perrin S. Confirmatory factor analysis of the Evidence-Based Practice Attitude Scale in a large and representative sample of Child and Adolescent Mental Health practitioners: Is the use of a total scale score justified?: researchsquare.com; 2019.

9. Ashcraft RG, Foster SL, Lowery AE, Henggeler SW, Chapman JE, Rowland MD. Measuring practitioner attitudes toward evidence-based treatments: A validation study. Journal of Child & Adolescent Substance Abuse. 2011;20(2):166-83.

10. Baumann AA, Vazquez AL, Macchione AC, Lima A, Coelho AF, Juras M, et al. Translation and validation of the evidence-based practice attitude scale (EBPAS-15) to Brazilian Portuguese: Examining providers' perspective about evidence-based parent intervention. Children & Youth Services Review. 2022;136.

11. Ayhan Baser D, Agadayi E, Gonderen Cakmak S, Kahveci R. Adaptation of the evidence-based practices attitude scale-15 in Turkish family medicine residents. International Journal of Clinical Practice. 2021;75(8):e14354.

12. Van Giang N, Lin SY, Thai DH. A psychometric evaluation of the Vietnamese version of the Evidence-Based Practice Attitudes and Beliefs Scales. International Journal of Nursing Practice. 2021;27(6):e12896.

13. Aarons GA, Cafri G, Lugo L, Sawitzky A. Expanding the domains of attitudes towards evidence-based practice: the evidence based practice attitude scale-50. Adm Policy Ment Health. 2012;39(5):331-40.

14. Yildiz D, Fidanci BE, Acikel C, Kaygusuz N, Yildirim C. Evaluating the Properties of the Evidence-Based Practice Attitude Scale (EBPAS-50) in Nurses in Turkey. International Journal of Caring Sciences. 2018;11(2):768-75.

15. Rye M, Torres EM, Friborg O, Skre I, Aarons GA. The Evidence-based Practice Attitude Scale-36 (EBPAS-36): a brief and pragmatic measure of attitudes to evidence-based practice validated in US and Norwegian samples. Implementation science : IS. 2017;12(1):44.

16. Szota K, Thielemann JFB, Christiansen H, Rye M, Aarons GA, Barke A. Cross-cultural adaption and psychometric investigation of the German version of the Evidence Based Practice Attitude Scale (EBPAS-36D). Health Research Policy & Systems. 2021;19(1):90.

17. Upton D, Upton P. Development of an evidence-based practice questionnaire for nurses. J Adv Nurs. 2006;53(4):454-8.

18. Son Y-J, Song Y, Park S-Y, Kim J-I. A psychometric evaluation of the Korean version of the evidence-based practice questionnaire for nurses. Contemp Nurse. 2014;49(1):4-14.

19. Tomotaki A, Fukahori H, Sakai I, Kurokohchi K. The development and validation of the Evidence-Based Practice Questionnaire: Japanese version. Int J Nurs Pract. 2018;24(2):e12617.

20. Yang R, Guo JW, Beck SL, Jiang F, Tang S. Psychometric Properties of the Chinese Version of the Evidence-Based Practice Questionnaire for Nurses. J Nurs Meas. 2019;27(3):E117-E31.

21. Zaybak A, Gunes UY, Dikmen Y, Arslan GG. Cultural Validation of the Turkish Version of Evidence-Based Practice Questionnaire. International Journal of Caring Sciences. 2017;10(1):37-46.

22. Sese-Abad A, De Pedro-Gomez J, Bennasar-Veny M, Sastre P, Fernandez-Dominguez JC, Morales-Asencio JM. A multisample model validation of the evidence-based practice questionnaire. Res Nurs Health. 2014;37(5):437-46.

23. Rospendowiski K, Alexandre NMC, Cornello ME. Cultural adaptation to Brazil and psychometric performance of the "Evidence-Based Practice Questionnaire". Acta Paulista De Enfermagem. 2014;27(5):405-11.

24. Pereira RP, Guerra AC, Cardoso MJ, dos Santos AT, de Figueiredo Mdo C, Carneiro AC. Validation of the Portuguese version of the Evidence-Based Practice Questionnaire. Rev Lat Am Enfermagem. 2015;23(2):345-51.

25. Fajarini M, Rahayu S, Setiawan A. The indonesia version of evidence-based practice questionnaire (EBPQ): Translation and Reliability. The 6th Padjadjaran …. 2021.

26. Melnyk BM, Fineout-Overholt E, Mays MZ. The evidence-based practice beliefs and implementation scales: psychometric properties of two new instruments. Worldviews Evid Based Nurs. 2008;5(4):208-16.

27. Grønvik CKU, Ødegård A, Bjørkly S. Factor Analytical Examination of the Evidence-Based Practice Beliefs Scale: Indications of a Two-Factor Structure: scirp.org; 2016.

28. Kerwien-Jacquier E, Verloo H, Pereira F, Peter KA. Adaptation and validation of the evidence-based practice beliefs and implementation scales into German. Nursing Open. 2020:12.

29. Thorsteinsson HS. Translation and validation of two evidence-based nursing practice instruments. Int Nurs Rev. 2012;59(2):259-65.

30. Verloo H, Desmedt M, Morin D. Adaptation and validation of the Evidence-Based Practice Belief and Implementation scales for French-speaking Swiss nurses and allied healthcare providers. J Clin Nurs. 2017;26(17-18):2735-43.

31. Melnyk BM, Hsieh AP, Gallagher-Ford L, Thomas B, Guo J, Tan A, et al. Psychometric Properties of the Short Versions of the EBP Beliefs Scale, the EBP Implementation Scale, and the EBP Organizational Culture and Readiness Scale. Worldviews on Evidence-Based Nursing. 2021;18(4):243-50.

32. Moore JL, Friis S, Graham ID, Gundersen ET, Nordvik JE. Reported use of evidence in clinical practice: a survey of rehabilitation practices in Norway. BMC Health Serv Res. 2018;18(1):379.

33. Dessie G, Jara D, Alem G, Mulugeta H, Zewdu T, Wagnew F, et al. Evidence-Based Practice and Associated Factors Among Health Care Providers Working in Public Hospitals in Northwest Ethiopia During 2017. Current Therapeutic Research, Clinical & Experimental. 2020;93:100613.

34. Al Zoubi F, Mayo N, Rochette A, Thomas A. Applying modern measurement approaches to constructs relevant to evidence-based practice among Canadian physical and occupational therapists. Implementation Science. 2018;13(1):152.

35. Bernal G, Rodriguez-Soto Ndel C. Development and psychometric properties of the evidence-based professional practice scale (EBPP-S). P R Health Sci J. 2010;29(4):385-90.

36. Jette DU, Bacon K, Batty C, Carlson M, Ferland A, Hemingway RD, et al. Evidence-based practice: beliefs, attitudes, knowledge, and behaviors of physical therapists. Phys Ther. 2003;83(9):786-805.

37. Ferreira RM, Ferreira PL, Cavalheiro L, Duarte JA, Gonçalves RS. Evidence-based practice questionnaire for physical therapists: Portuguese translation, adaptation, validity, and reliability. Journal of Evidence-Based Healthcare. 2019;1(2):83-98.

38. Bernhardsson S, Larsson ME. Measuring evidence-based practice in physical therapy: translation, adaptation, further development, validation, and reliability test of a questionnaire. Phys Ther. 2013;93(6):819-32.

39. Kaper NM, Swennen MH, van Wijk AJ, Kalkman CJ, van Rheenen N, van der Graaf Y, et al. The "evidence-based practice inventory": reliability and validity was demonstrated for a novel instrument to identify barriers and facilitators for Evidence Based Practice in health care. J Clin Epidemiol. 2015;68(11):1261-9.

40. Braun T, Ehrenbrusthoff K, Bahns C, Happe L, Kopkow C. Cross-cultural adaptation, internal consistency, test-retest reliability and feasibility of the German version of the evidence-based practice inventory. BMC Health Serv Res. 2019;19(1):455.

41. Salbach NM, Jaglal SB, Williams JI. Reliability and validity of the evidence-based practice confidence (EPIC) scale. J Contin Educ Health Prof. 2013;33(1):33-40.

42. Clyde JH, Brooks D, Cameron JI, Salbach NM. Validation of the Evidence-Based Practice Confidence (EPIC) Scale With Occupational Therapists. Am J Occup Ther. 2016;70(2):7002280010p1-9.

43. Borntrager CF, Chorpita BF, Higa-McMillan C, Weisz JR. Provider attitudes toward evidence-based practices: are the concerns with the evidence or with the manuals? Psychiatr Serv. 2009;60(5):677-81.

44. Park H, Ebesutani CK, Chung KM, Stanick C. Cross-Cultural Validation of the Modified Practice Attitudes Scale: Initial Factor Analysis and a New Factor Model. Assessment. 2018;25(1):126-38.

45. Rubin A, Parrish DE. Development and validation of the Evidence-based Practice Process Assessment Scale: Preliminary findings. Research on Social Work Practice. 2010;20(6):629-40.

46. Rubin A, Parrish DE. Validation of the evidence-based practice Process Assessment Scale. Research on Social Work Practice. 2011;21(1):106-18.

47. Parrish DE, Rubin A. Validation of the Evidence-Based Practice Process Assessment Scale-Short Version. Research on Social Work Practice. 2011;21(2):200-11.

48. Chang AM, Crowe L. Validation of scales measuring self-efficacy and outcome expectancy in evidence-based practice. Worldviews Evid Based Nurs. 2011;8(2):106-15.

49. Oh EG, Yang YL, Sung JH, Park CG, Chang AM. Psychometric Properties of Korean Version of Self-Efficacy of Evidence-Based Practice Scale. Asian Nurs Res (Korean Soc Nurs Sci). 2016;10(3):207-12.

50. Tucker SJ, Olson ME, Frusti DK. Evidence-Based Practice Self-efficacy Scale Preliminary Reliability and Validity. Clin Nurse Spec. 2009;23(4):207-15.

51. Wallin L, Bostrom AM, Gustavsson JP. Capability beliefs regarding evidence-based practice are associated with application of EBP and research use: validation of a new measure. Worldviews Evid Based Nurs. 2012;9(3):139-48.

52. Sleutel MR, Barbosa-Leiker C, Wilson M. Psychometric Testing of the Health Care Evidence-Based Practice Assessment Tool. J Nurs Meas. 2015;23(3):485-98.

53. Shi Q, Chesworth BM, Law M, Haynes RB, MacDermid JC. A modified evidence-based practice- knowledge, attitudes, behaviour and decisions/outcomes questionnaire is valid across multiple professions involved in pain management. BMC Med Educ. 2014;14:263.

54. Connor L, Paul F, McCabe M, Ziniel S. Measuring Nurses' Value, Implementation, and Knowledge of Evidence-Based Practice: Further Psychometric Testing of the Quick-EBP-VIK Survey. Worldviews Evid Based Nurs. 2017;14(1):10-21.

55. Zhou C, Wang Y, Wang S, Ou J, Wu Y. Translation, cultural adaptation, validation, and reliability study of the Quick-EBP-VIK instrument: Chinese version. J Eval Clin Pract. 2019;25(5):856-63.

56. Fernandez-Dominguez JC, de Pedro-Gomez JE, Morales-Asencio JM, Bennasar-Veny M, Sastre-Fullana P, Sese-Abad A. Health Sciences-Evidence Based Practice questionnaire (HS-EBP) for measuring transprofessional evidence-based practice: Creation, development and psychometric validation. PLoS ONE [Electronic Resource]. 2017;12(5):e0177172.

57. Patelarou AE, Dafermos V, Brokalaki H, Melas CD, Koukia E. The evidence-based practice readiness survey: a structural equation modeling approach for a Greek sample. International Journal of Evidence-Based Healthcare. 2015;13(2):77-86.

58. McEvoy MP, Williams MT, Olds TS. Development and psychometric testing of a trans-professional evidence-based practice profile questionnaire. Med Teach. 2010;32(9):e373-80.

59. Hu MY, Wu YN, McEvoy MP, Wang YF, Cong WL, Liu LP, et al. Development and validation of the Chinese version of the evidence-based practice profile questionnaire (EBP<sup>2</sup>Q). BMC Med Educ. 2020;20(1):280.

60. Panczyk M, Belowska J, Zarzeka A, Samolinski L, Zmuda-Trzebiatowska H, Gotlib J. Validation study of the Polish version of the Evidence-Based Practice Profile Questionnaire. BMC Med Educ. 2017;17(1):38.

61. Titlestad KB, Snibsoer AK, Stromme H, Nortvedt MW, Graverholt B, Espehaug B. Translation, cross-cultural adaption and measurement properties of the evidence-based practice profile. BMC Res Notes. 2017;10(1):44-.

62. Belowska J, Panczyk M, Zarzeka A, Iwanow L, Cieslak I, Gotlib J. Promoting evidence-based practice - perceived knowledge, behaviours and attitudes of Polish nurses: a cross-sectional validation study. International Journal of Occupational Safety & Ergonomics. 2020;26(2):397-405.

63. Mah AC, Hill KA, Cicero DC, Nakamura BJ. A Psychometric Evaluation of the Intention Scale for Providers-Direct Items. J Behav Health Serv Res. 2020;47(2):245-63.

64. Ruzafa-Martinez M, Lopez-Iborra L, Madrigal-Torres M. Attitude towards Evidence-Based Nursing Questionnaire: development and psychometric testing in Spanish community nurses. J Eval Clin Pract. 2011;17(4):664-70.

65. Diermayr G, Schachner H, Eidenberger M, Lohkamp M, Salbach NM. Evidence-based practice in physical therapy in Austria: Current state and factors associated with EBP engagement. J Eval Clin Pract. 2015;21(6):1219-34.

66. Ruzafa-Martinez M, Fern, ez-Salazar S, Leal-Costa C, Ramos-Morcillo AJ. Questionnaire to Evaluate the Competency in Evidence-Based Practice of Registered Nurses (EBP-COQ Prof©): Development and Psychometric Validation. Worldviews on Evidence-Based Nursing. 2020;17(5):366-75.

67. Schetaki S, Patelarou E, Giakoumidakis K, Trivli A, Kleisiaris C, Patelarou A. Translation and Validation of the Greek Version of the Evidence-Based Practice Competency Questionnaire for Registered Nurses (EBP-COQ Prof©). Nursing Reports. 2022;12(4):693-707.

68. Ruano ASM, Motter FR, Lopes LC. Design and validity of an instrument to assess healthcare professionals' perceptions, behaviour, self-efficacy and attitudes towards evidence-based health practice: I-SABE. BMJ Open. 2022;12.

69. Norhayati MN, Nawi ZM. Validity and reliability of the Noor Evidence-Based Medicine Questionnaire: A cross-sectional study. PLoS ONE [Electronic Resource]. 2021;16(4):e0249660.

70. Abuadas MH, Albikawi ZF, Abuadas F. Development and Validation of Questionnaire Measuring Registered Nurses' Competencies, Beliefs, Facilitators, Barriers, and Implementation of Evidence-Based Practice (EBP-CBFRI). Journal of Nursing Measurement. 2021;13:13.

71. Prinsen CAC, Mokkink LB, Bouter LM, Alonso J, Patrick DL, de Vet HCW, et al. COSMIN guideline for systematic reviews of patient-reported outcome measures. Qual Life Res. 2018;27(5):1147-57.

72. Elsman EBM, Mokkink LB, Langendoen-Gort M, Rutters F, Beulens J, Elders PJM, et al. Systematic review on the measurement properties of diabetes-specific patient-reported outcome measures (PROMs) for measuring physical functioning in people with type 2 diabetes. BMJ Open Diabetes Res Care. 2022;10(3).
